# A review of glycosylation diversity and biosynthetic advances of steroidal saponins in *Paris polyphylla* var. *yunnanensis*

**DOI:** 10.3389/fpls.2026.1905483

**Published:** 2026-08-12

**Authors:** Yueyan Luo, Ran Guo, Xinyan He, Anzhong Peng, Longxing Li, Haifeng Li

**Affiliations:** 1 College of Pharmacy & Yunnan Key Laboratory of Screening and Research on Anti-pathogenic Plant Resources from Western Yunnan, Dali University, Dali, Yunnan, China; 2 Analysis and Testing Center, Dali University, Dali, Yunnan, China

**Keywords:** *Paris polyphylla* var. *yunnanensis*, steroidal saponins, glycosylation patterns, biosynthetic pathways, sustainable utilization

## Abstract

Steroidal saponins are the principal bioactive constituents of *Paris polyphylla* var. *yunnanensis* (*P*. *polyphylla*) and are widely used in traditional medicine and the pharmaceutical industry. However, increasing market demand, combined with the species slow growth cycle, has led to severe depletion of wild resources, while the natural abundance of key pharmacologically active compounds remains relatively low. Recent advances in multiomics technologies and synthetic biology have progressively clarified the biosynthetic pathways of steroidal saponins in *P*. *polyphylla*. Consequently, improving the production of these valuable metabolites through efficient heterologous biosynthesis has become an important strategy for their sustainable utilization. In this review, we systematically summarize the steroidal saponins identified in *P*. *polyphylla*, with particular emphasis on their glycosylation patterns, and comprehensively discuss recent progress in elucidating their biosynthetic pathways. This review aims to elucidate the structural diversity and glycosylation patterns of steroidal saponins in *P*. *polyphylla* and to systematically summarize their biosynthetic pathways, including steroidal backbone formation and glycosylation processes. By integrating recent advances in the chemical characterization and biosynthetic mechanisms of steroidal saponins, this review provides insights into the molecular basis underlying their structural diversity and establishes a theoretical framework for the sustainable utilization of *P*. *polyphylla*.

## 1 Introduction

**Figure 1:**
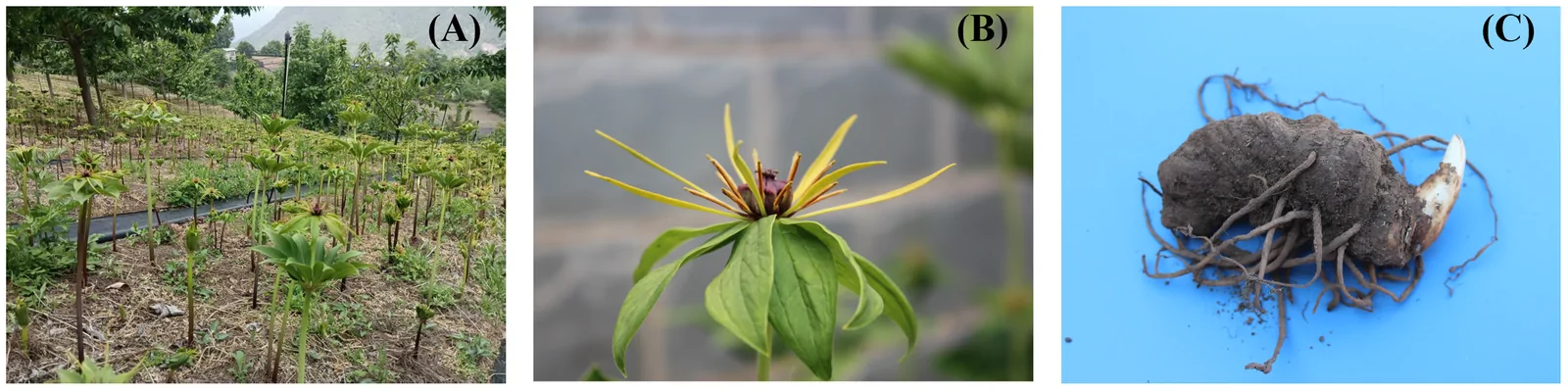
Morphology of *P*. *polyphylla*. **(A)** Whole plant; **(B)** Flower; **(C)** Rhizome.

*Paris polyphylla* var. *yunnanensis* (*P*. *polyphylla*) is a perennial herbaceous plant of the genus *Paris* in the family Melanthiaceae, commonly known as “Dujiaolian” (**Figure 1**). It is recognized in Pharmacopoeia of People’s Republic of China as an authentic source of the traditional Chinese medicinal material Rhizoma Paridis (Commission C.P, 2025), and is mainly distributed in Yunnan, Guizhou, and Sichuan provinces of China (Zhao et al., 2014). As one of the “Top Ten Yunnan Medicinal Herbs”, *P*. *polyphylla* has been widely utilized in both traditional practices and the pharmaceutical industry. It is characterized by therapeutic properties such as clearing heat and detoxifying, reducing swelling and relieving pain, and calming the liver to alleviate convulsions. In folk medicine, it has also been commonly used for the treatment of conditions including pulmonary tuberculosis–related cough, asthma, rheumatic pain, and tumors (Yan et al., 2021). Modern studies have demonstrated that *P*. *polyphylla* contains a diverse array of phytochemicals, including steroidal saponins, cholestane-type compounds, phytosterols, and flavonoids (Li and Du, 2023). Among these, steroidal saponins are recognized as the principal bioactive constituents, exhibiting a broad spectrum of pharmacological activities, such as antitumor, antimyocardial ischemia, and anti-inflammatory as well as analgesic effects (Ding et al., 2021; Wang et al., 2023; Wu et al., 2017). With the growing recognition of the medicinal value of Rhizoma Paridis and the continuous discovery of its new therapeutic potentials, market demand for this medicinal material has increased substantially. Driven by economic interests, indiscriminate harvesting and overexploitation have not been fundamentally curbed, leading to the near depletion of wild resources. Meanwhile, *P*. *polyphylla* is characterized by a long growth cycle, complex composition of medicinal constituents, and extremely slow accumulation of bioactive compounds. It typically requires 8–10 years from germplasm germination to reach a stage suitable for medicinal use. Although considerable progress has been made in artificial cultivation and tissue culture technologies, improving the accumulation efficiency of pharmacologically important steroidal saponins remains a major challenge.

Steroidal saponins are generally composed of two structural components: a steroidal aglycone and one or more sugar chains. Structural modifications of the aglycone scaffold, together with the composition, number, and glycosidic linkage patterns of the sugar moieties, contribute to their remarkable structural diversity. Among these structural features, the glycosylation pattern is not only closely associated with the structural diversity of steroidal saponins but also serves as one of the major determinants of their physicochemical properties and biological activities (Shin and Oh, 2023; Zhang et al., 2022). However, current studies on steroidal saponins from *P*. *polyphylla* have primarily focused on the identification of individual compounds and the evaluation of their pharmacological activities, whereas systematic characterization of their glycosylation patterns and structural features remains relatively limited.

In recent years, with the rapid advancement of biosynthetic technologies and heterologous expression systems, significant progress has been made in the study of steroidal saponin biosynthesis in *P*. *polyphylla*. A series of key enzymes involved in this pathway–from upstream backbone construction to downstream glycosylation modifications–have been successfully identified, laying a solid foundation for elucidating the complete biosynthetic network. At the same time, researchers have systematically elucidated the biosynthetic pathway and established efficient routes for the production of steroidal saponins, thereby laying a solid foundation for the heterologous biosynthesis of steroidal saponin compounds (Xu B. et al., 2024, Xu W. K. et al., 2024; Yin et al., 2023). However, current studies on the glycosylation patterns and biosynthesis of steroidal saponins in *P*. *polyphylla* remain fragmented, and a comprehensive synthesis of the available evidence is still lacking, thereby limiting a systematic understanding of their structural diversification and biosynthetic mechanisms.

Therefore, this paper provides a systematic review of the structural classification and glycosidic linkage patterns of steroidal saponins in *P*. *polyphylla*, and comprehensively summarizes the key enzymes and regulatory mechanisms involved in their biosynthetic pathways. It aims to provide a comprehensive reference for elucidating the molecular basis underlying the structural diversity and biosynthetic regulatory network of steroidal saponins in *P*. *polyphylla*, thereby facilitating future studies on the exploitation of these bioactive compounds, the functional characterization of key biosynthetic enzymes, and the elucidation of metabolic regulatory mechanisms.

## 2 Methodology

A comprehensive literature search was conducted using bibliographic databases, including Web of Science, PubMed, ScienceDirect, CAS SciFinder, Google Scholar, China National Knowledge Infrastructure (CNKI), and Wanfang Data, to retrieve relevant publications published between 1960 to 2025. The literature search was performed using combinations of the following keywords: “*Paris polyphylla* var. *yunnanensis*”, “*Paris polyphylla*”, “*Paris yunnanensis*”, “steroidal saponins”, “polyphyllins”, “glycosylation”, “biosynthesis”, “cytochrome P450 (CYP450)”, “UDP-glycosyltransferase”, “pharmacology”, and “synthetic biology”.

Approximately 115 publications were initially screened, and the most relevant studies were selected for inclusion in this review. The selected literature mainly focused on the taxonomy, steroidal saponin constituents, biosynthetic pathways, and glycosylation processes of *P*. *polyphylla*. Conference abstracts, duplicate publications, studies unrelated to steroidal saponins in *P*. *polyphylla*, and reports lacking sufficient experimental evidence were excluded.

The accepted scientific name, synonyms, and distribution information of *P*. *polyphylla* were verified using Plants of the World Online (POWO), World Flora Online (WFO), Flora of China, and the Chinese Pharmacopoeia. The chemical structures of steroidal saponins were verified using the PubChem database and drawn using ChemDraw 23.1.1.

## 3 Overview of *P. polyphylla*

### 3.1 Botanical characteristics and taxonomy

*Paris polyphylla* var. *yunnanensis* (Franch.) Hand.-Mazz. is a perennial medicinal herb belonging to the genus *Paris* in the family Melanthiaceae. Morphologically, it is characterized by a stout rhizome, an erect unbranched stem, whorled leaves, a solitary terminal flower, and a capsule fruit. The leaves are lanceolate, ovate-oblong, or obovate-lanceolate in shape (Liang and Soukup, 2000). The rhizome is the principal medicinal organ and serves as the primary site for the accumulation of bioactive secondary metabolites. Rather than being regarded as a weed species, *P*. *polyphylla* is recognized as a medicinal plant resource of considerable pharmacological importance owing to its unique phytochemical composition and long-standing medicinal value. In addition, its distinctive plant architecture, characteristic whorled phyllotaxy, and ornamental flowers confer considerable horticultural and ornamental potential.

In traditional classification systems, the genus *Paris* was placed within the family Liliaceae. However, with advances in molecular phylogenetic studies, it has been reassigned to the family Melanthiaceae. Accordingly, its taxonomic hierarchy can be summarized as follows: Kingdom Plantae, Tracheophytes, Angiosperms, Monocots, Order Liliales, Family Melanthiaceae, Genus *Paris*, Species *Paris polyphylla*, and Variety *Paris polyphylla* var. *yunnanensis (*Ji, 2021*)*. The genus *Paris* comprises approximately 26 species. Within the *Paris polyphylla* species complex, *P*. *polyphylla* var. yunnanensis is an important medicinal taxon and serves as one of the authentic botanical sources of *Rhizoma Paridis*, the official herbal medicine recorded in the Pharmacopoeia of the People’s Republic of China (Commission C.P, 2025; Ji, 2021).The taxonomy of the genus Paris has undergone multiple revisions, resulting in various historical nomenclatural treatments and taxonomic interpretations. Consequently, different contemporary taxonomic databases do not adopt a fully consistent classification for this taxon. The Pharmacopoeia of the People’s Republic of China and Flora of China continue to recognize *Paris polyphylla* var. *yunnanensis* as the accepted name (Commission C.P, 2025; Liang and Soukup, 2000), whereas Plants of the World Online (POWO) and World Flora Online (WFO) currently recognize *Paris yunnanensis* Franch. as the accepted name and treat *P*. *polyphylla* var. *yunnanensis* as its synonym (Plants of the World Online, 2026; World Flora Online, 2026).

### 3.2 Geographical distribution and ecological characteristics

Species of the genus *Paris* are widely distributed throughout the temperate and subtropical regions of Asia, occurring predominantly in China, the Himalayan region, and neighboring countries, including Nepal, Bhutan, northern India, Myanmar, and Vietnam (Sharma et al., 2015). In contrast, the distribution of *P*. *polyphylla* is relatively restricted and is primarily confined to southwestern China, particularly Yunnan, Guizhou, and Sichuan provinces (Commission C.P, 2025). This species typically inhabits cool, shaded, and humid environments and is commonly found in forest understories or along roadsides at elevations of approximately 1,400-3,600 m (Plants of the World Online, 2026). Owing to its prolonged life cycle, *P*. *polyphylla* generally requires 8–10 years to reach medicinal maturity from seed germination. Combined with long-term overharvesting and habitat disturbance, these biological characteristics have resulted in a continuous decline of wild populations. Consequently, the species has been listed as a National Key Protected Wild Plant (Class II) in China.

### 3.3 Traditional medicinal uses and regional applications

As an important medicinal plant, *P*. *polyphylla* has been used in traditional Chinese medicine for several centuries. Its rhizome is traditionally prescribed for its heat-clearing and detoxifying, swelling-reducing and analgesic, as well as liver-calming and anticonvulsant properties. It has long been used to treat inflammatory disorders, sore throat, carbuncles and abscesses, traumatic injuries, and other related conditions (Commission C.P, 2025). In addition, in traditional folk medicine, the dried rhizomes are commonly processed by decoction, honey-processing, or alcohol maceration, and are primarily used for the treatment of inflammation, abscesses, wounds, snake bites, febrile illnesses, and as an adjunctive therapy for tumors (Yan et al., 2024). In the Himalayan region and parts of Southeast Asia, species of the genus *Paris* have also been traditionally employed for detoxification, wound healing, the management of inflammatory conditions, and the treatment of infectious diseases (Kunwar et al., 2020; Sharma et al., 2015). Modern pharmacological studies have demonstrated that steroidal saponins are the principal bioactive constituents responsible for the therapeutic activities of *P*. *polyphylla*. In particular, polyphyllin I, II, VI, and VII, together with other diosgenin-type and pennogenin-type steroidal saponins, have exhibited diverse pharmacological activities in experimental models, including anti-inflammatory, hemostatic, antibacterial, cytotoxic, and apoptosis-inducing effects (Yan et al., 2021, 2023).

## 4 Chemical constituents

### 4.1 Structural characteristics of steroidal saponins in *P*. *polyphylla*

**Figure 2:**
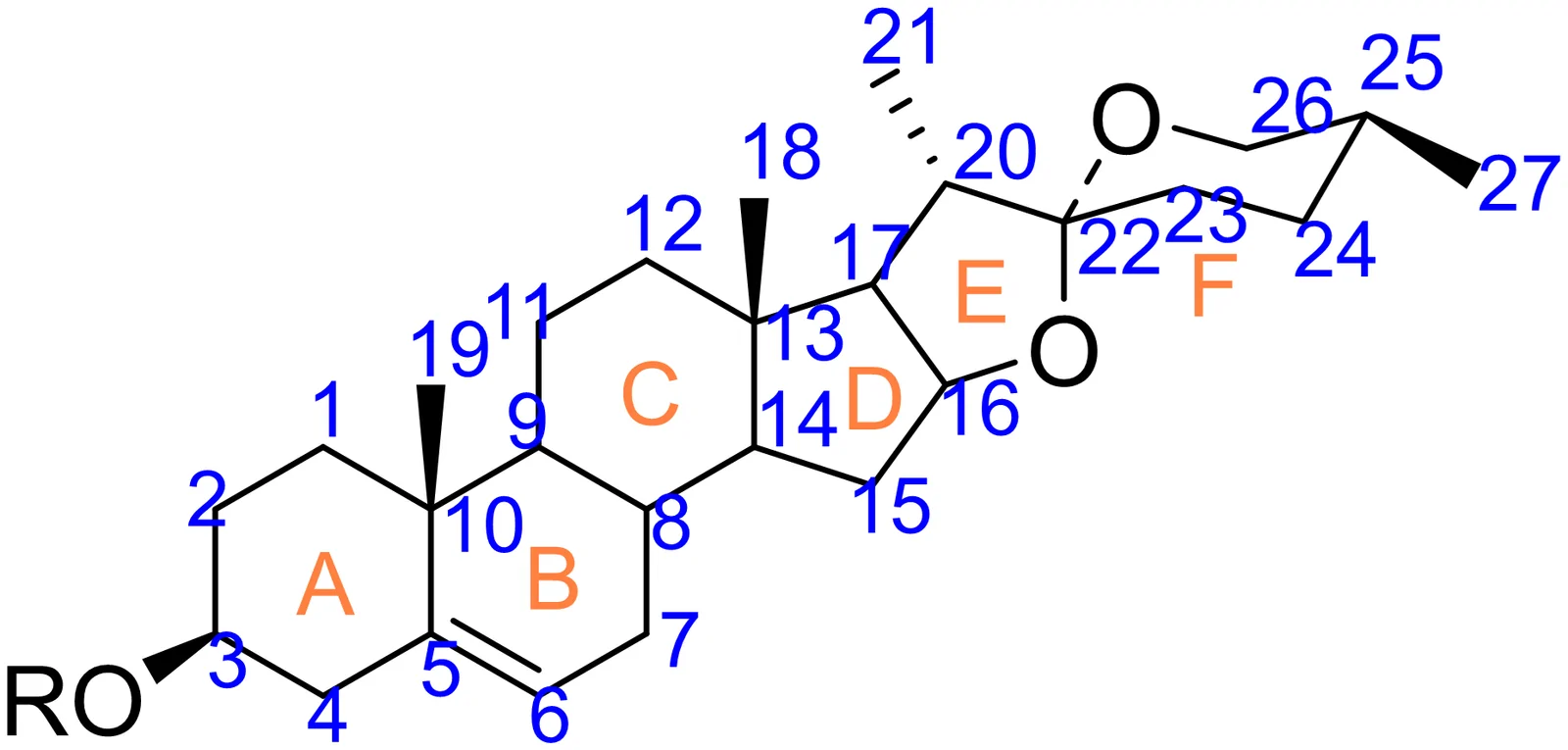
Representative molecular skeletons of steroidal saponins.

*P*. *polyphylla* is rich in secondary metabolites, among which steroidal saponins constitute the primary bioactive components. These compounds are hexacyclic molecules containing 27 carbon atoms, structurally composed of a hydrophobic aglycone and hydrophilic sugar moieties. The aglycone core consists of four fused rings, designated as A, B, C, and D, forming a hydrogenated cyclopentanophenanthrene framework, while the E and F rings are connected through a spiroketal linkage (**Figure 2**) (Moses et al., 2014; Wang et al., 2024). This study systematically compiles 122 steroidal saponins that have been isolated and identified from *P*. *polyphylla* as reported in the literature. These compounds are generally extracted using organic solvents, primarily ethanol or methanol, followed by liquid–liquid partitioning and purification through various chromatographic techniques. The stationary phases commonly employed include silica gel, macroporous adsorption resins, reversed-phase chromatographic materials, and preparative high-performance liquid chromatography (prep-HPLC). Structural elucidation mainly relies on spectroscopic techniques, particularly nuclear magnetic resonance (NMR) spectroscopy and mass spectrometry (MS). In recent years, liquid chromatography–tandem mass spectrometry (LC–MS/MS)-based approaches have been increasingly applied for the rapid identification and profiling of steroidal saponins. According to the structural characteristics of their aglycone skeletons, these steroidal saponins can be classified into several major groups, including diosgenin-type saponins, protodioscin-type saponins, pseudoprotodioscin-type saponins, pennogenin-type saponins, isonuatigenin-type saponins, nuatigenin-type saponins, and other types. Among these, diosgenin-type and pennogenin-type saponins are considered the major bioactive constituents of steroidal saponins in *P*. *polyphylla* (Rawat et al., 2023b; Zhang et al., 2024; Zhu et al., 2019).

**Figure 3:**
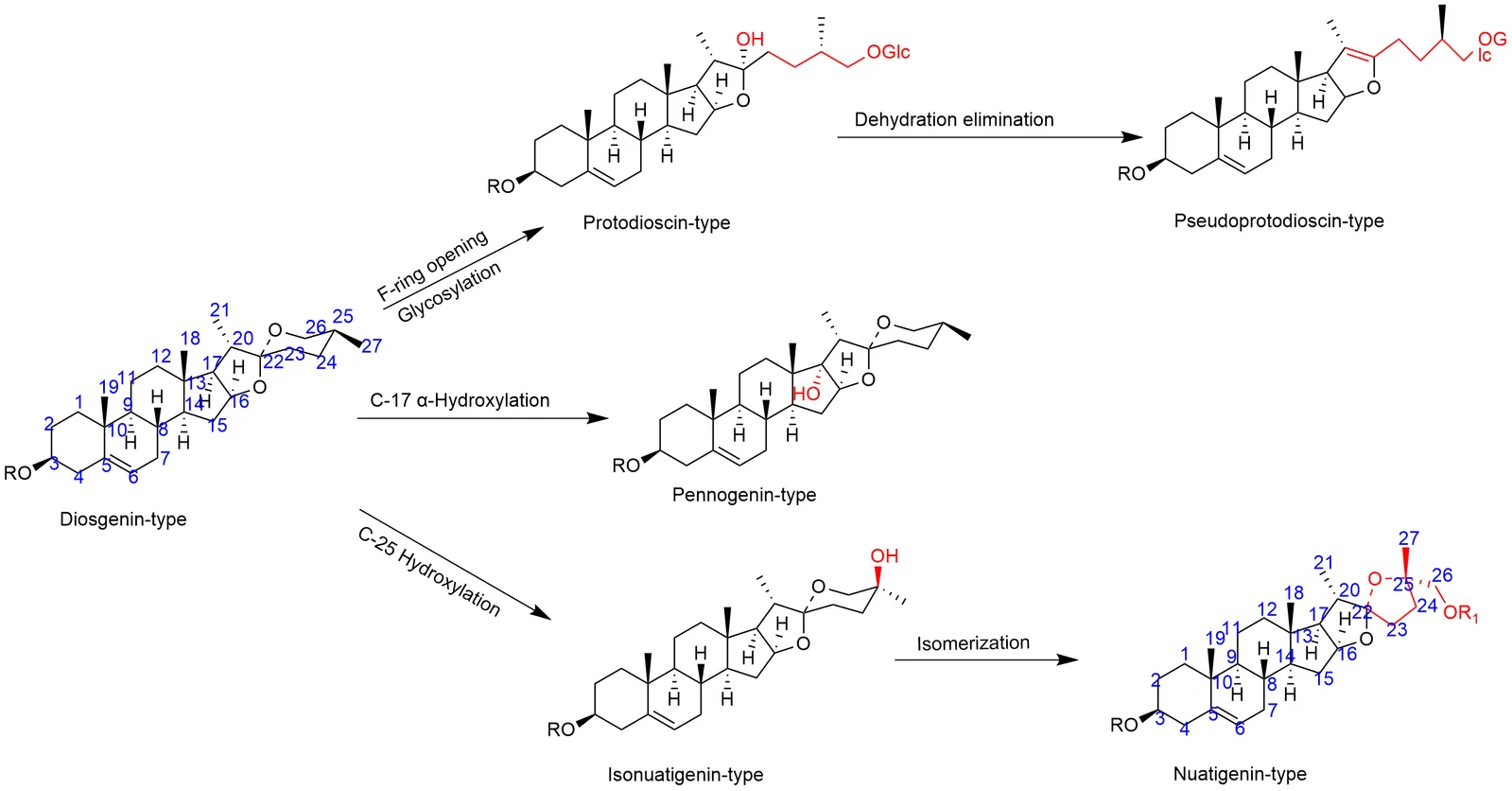
Structural classification of steroidal saponins and the relationships among different structural types.

Diosgenin plays a pivotal role in the biosynthesis of various steroidal saponins (**Figure 3**). (1) Diosgenin can undergo F-ring opening, hydroxylation, and glycosylation reactions to form the basic skeleton of protodioscin-type compounds, which are subsequently diversified into different protodioscin-type saponins through further glycosylation modifications. Thereafter, protodioscin-type compounds can undergo dehydration elimination at the C-22 and C-23 positions to generate the aglycone backbone of pseudoprotodioscin-type saponins, which are then glycosylated to yield the corresponding pseudoprotodioscin-type saponins. (2) α-Hydroxylation at the C-17 position of diosgenin leads to the formation of the aglycone backbone of pennogenin-type compounds, which are subsequently diversified into various pennogenin-type saponins through glycosylation reactions. (3) Hydroxylation at the C-25 position of diosgenin leads to the formation of isonuatigenin, which can subsequently undergo isomerization to yield nuatigenin. These intermediates give rise to the structural diversity of the corresponding saponins (Huang et al., 2009).

### 4.2 Diversity of steroidal saponins in *P*. *polyphylla*

#### 4.2.1 Diosgenin-type saponins

**Figure 4:**
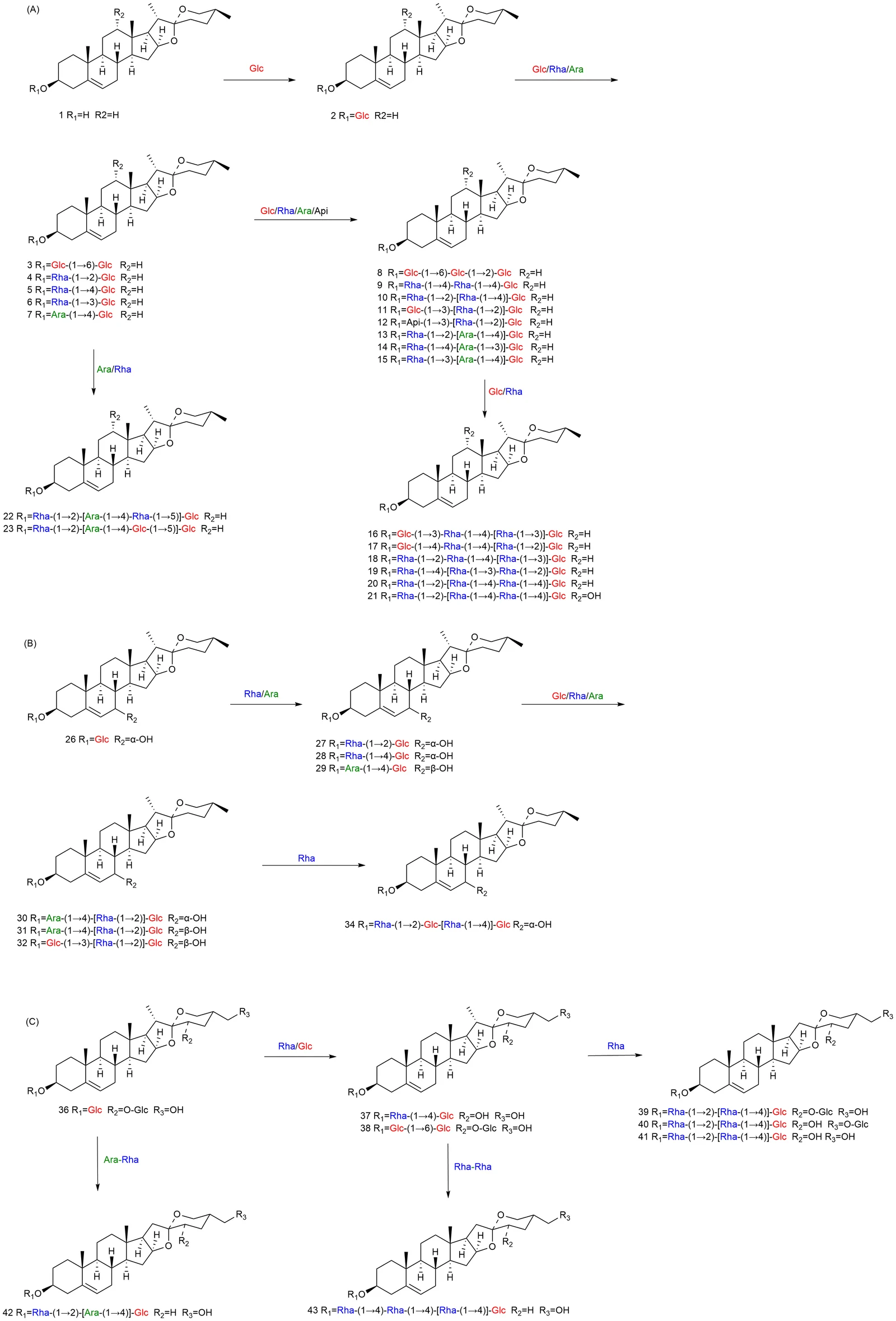
Glycosylation patterns of diosgenin-type saponin. **(A–C)** represent distinct skeletons of diosgenin-type saponins formed by substitutions at different positions on the aglycone core. Specifically, **(A)** denotes substitution at the C-3 and C-12 positions; **(B)** denotes substitution at the C-3 and C-7 positions; and **(C)** denotes substitution at the C-3, C-23, and C-27 positions.

A total of 48 diosgenin-type chemical constituents (1-48) have been identified in *P*. *polyphylla*. Structural modifications of these compounds mainly occur at the C-3, C-7, C-23, C-24, and C-27 positions. Among these, the C-3 position is predominantly substituted by sugar chains to form glycosidic linkages, representing the primary site of glycosylation, while the other positions are mainly substituted by hydroxyl groups. The sugar chains attached at the C-3 position are primarily composed of glucose (Glc), rhamnose (Rha), and arabinose (Ara), most commonly forming disaccharide, trisaccharide, or tetrasaccharide moieties through glycosylation. Within these glycan chains, Glc is typically directly linked to the aglycone attachment site, serving as the inner sugar, whereas the terminal sugar residues are predominantly Rha. The types of monosaccharides, their linkage sequences, and branching patterns within the sugar chains collectively contribute to the unique chemical characteristics of steroidal saponins. In disaccharide chains, the most common linkage pattern is Glc-Rha, as observed in com-pounds such as polyphyllin V, polyphyllin C, and sansevierin A. Trisaccharide and tetrasaccharide chains are generally formed by further extension or branching based on the Glc-Rha core structure (**Table 1**; **Figure 4**).

**Table 1:** Diosgenin-type saponins from *P. polyphylla*.

NO.	Aglycone skeleton	Substituent					Compound name
		R_1_		R_2_			
1	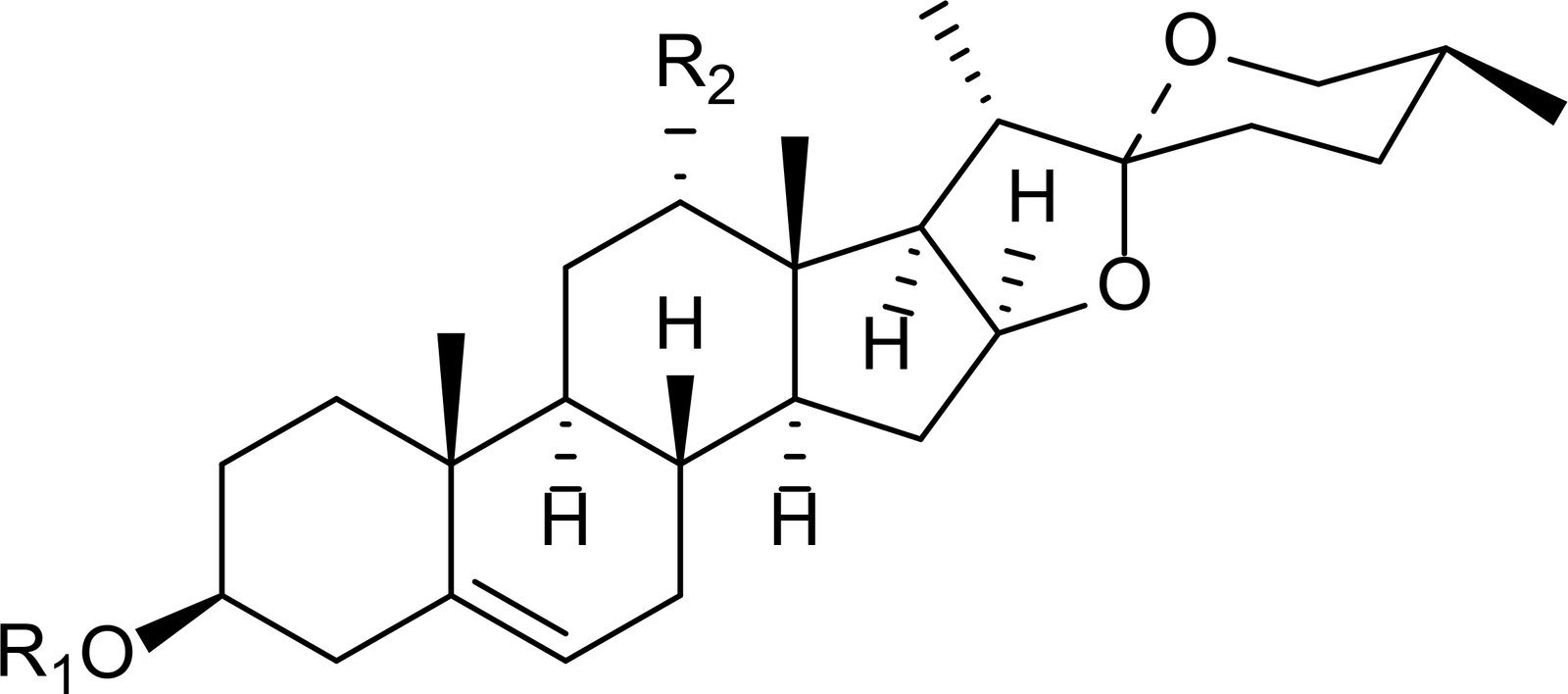	H		H			diosgenin (Huang and Zhou, 1962)
2		Glc		H			trillin (Nohara et al., 1973)
3		Glc-(1→6)-Glc		H			diosgenin-3-O-*β*-D-glucopyranosyl-(1→6)-*β*-D-glucopyranoside (Wu et al., 2012b)
4		Rha-(1→2)-Gl		H			polyphyllin V (Nohara et al., 1973; Wen et al., 2015a)
5		Rha-(1→4)-Glc		H			diosgenin-3-O-*α*-L-rhamnopyranosyl-(1→4)-*β*-D-glucopyranoside (Qin et al., 2012; Wang et al., 2007)
6		Rha-(1→3)-Glc		H			polyphyllin C (Chen and Zhou, 1981)
7		Ara-(1→4)-Glc		H			diosgenin-3-O-*α*-L-arabinofuranosyl-(1→4)-*β*-D-glucopyranoside (Chen and Zhou, 1987)
8		Glc-(1→6)-Glc-(1→2)-Glc		H			diosgenin-3-O-*β*-D-glucopyranosyl-(1→6)-*β*-D-glucopyranosyl-(1→2)-*β*-D-glucopyranoside (Wu et al., 2012b)
9		Rha-(1→4)-Rha-(1→4)-Glc		H			diosgenin-3-O-*α*-L-rhamnopyranosyl-(1→4)-*α*-L-rhamnopyranosyl-(1→4)-*β*-D-glucopyranoside (Wu et al., 2012b)
10		Rha-(1→2)-[Rha-(1→4)]-Glc		H			polyphyllin III (Qin et al., 2012; Singh et al., 1980)
11		Glc-(1→3)-[Rha-(1→2)]-Glc		H			gracillin (Kang et al., 2005)
12		Api-(1→3)-[Rha-(1→2)]-Glc		H			diosgenin-3-O-*α*-L-rhamnopyranosyl-(1→2)-[*α*-L-apiofuranosyl-(1→3)]-*β*-D-glucopyranoside (Wu et al., 2012a)
13		Rha-(1→2)-[Ara-(1→4)]-Glc		H			polyphyllin I (Singh et al., 1980)
14		Rha-(1→4)-[Ara-(1→3)]-Glc		H			diosgenin-3-O-*α*-L-rhamnopyranosyl-(1→4)-[*α*-L-arabinofuranosyl-(1→3)]-*β*-D-glucopyranoside (Wu et al., 2012a)
15		Rha-(1→3)-[Ara-(1→4)]-Glc		H			diosgenin-3-O-*α*-L-rhamnopyranosyl-(1→3)-[*α*-L-arabinofuranosyl-(1→4)]-*β*-D-glucopyranoside (Chen and Zhou, 1981)
16		Glc-(1→3)-Rha-(1→4)-[Rha-(1→3)]-Glc		H			pariphyllin A (Wei et al., 2014; Wu et al., 2012a)
17		Glc-(1→4)-Rha-(1→4)-[Rha-(1→2)]-Glc		H			diosgein-3-O-*β*-D-glucopyranosyl-(1→4)-*α*-L-rhamnopyranosyl-(1→4)-[*α*-L-rhamnopyranosyl-(1→2)]-*β*-D-glucopyranoside (Guan et al., 2019; Wei et al., 2014)
18		Rha-(1→2)-Rha-(1→4)-[Rha-(1→3)]-Glc		H			polyphyllin E (Chen and Zhou, 1981)
19		Rha-(1→4)-[Rha-(1→3)-Rha-(1→2)]-Glc		H			polyphyllin F (Chen and Zhou, 1981)
20		Rha-(1→2)-[Rha-(1→4)-Rha-(1→4)]-Glc		H			polyphyllin II (Qin et al., 2012; Singh et al., 1980)
21		Rha-(1→2)-[Rha-(1→4)-Rha-(1→4)]-Glc		OH			parisyunnanoside C (Zhao et al., 2009)
22		Rha-(1→2)-[Ara-(1→4)-Rha-(1→5)]-Glc		H			reclinatoside (Zhao et al., 2009)
23		Rha-(1→2)-[Ara-(1→4)-Glc-(1→5)]-Glc		H			loureiroside (Zhao et al., 2009)
24	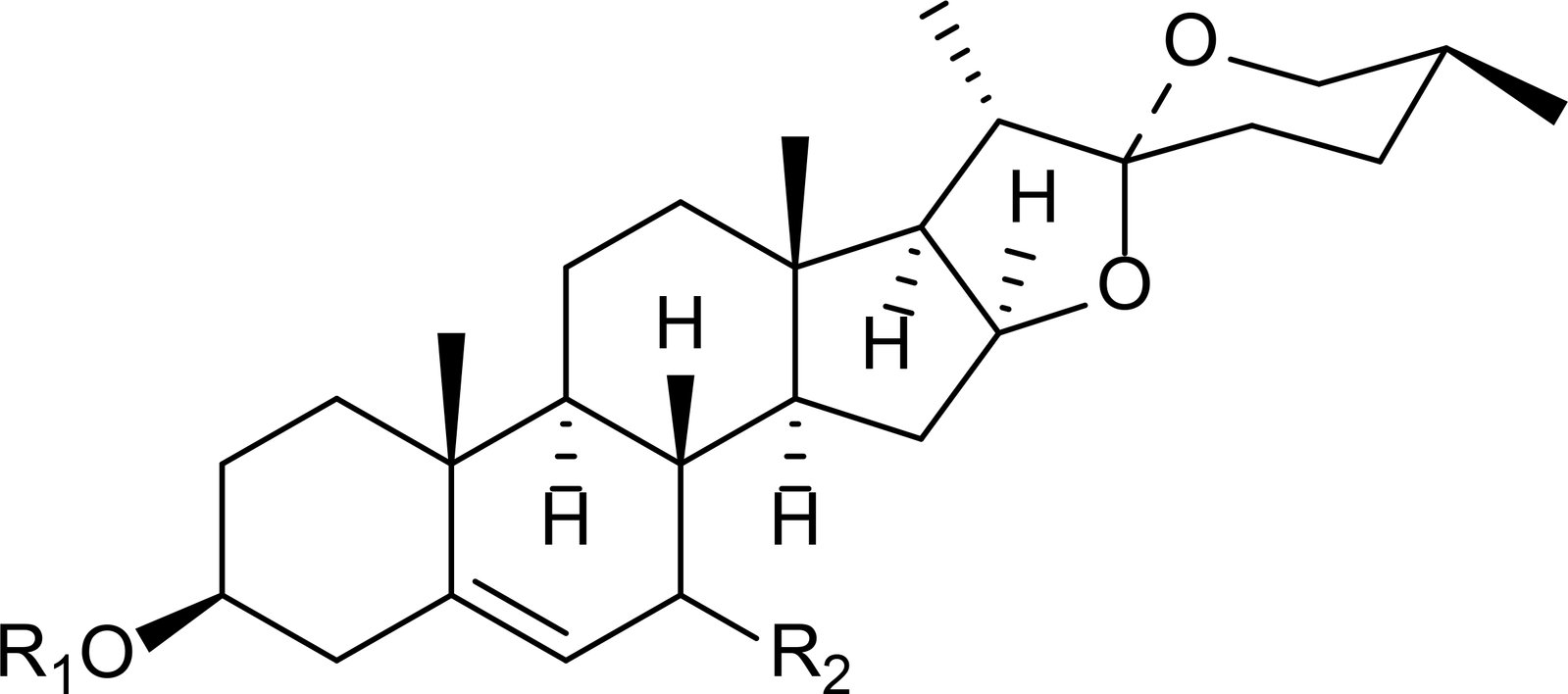	β-OH		β-OH			(25R)-spirost-5-en-3*β*, 7*β*-diol (Jiang et al., 2021)
25		β-OH		α-OH			(25R)-spirost-5-en-3*β*, 7*α*-diol (Jiang et al., 2021)
26		Glc		α-OH			chonglouoside SL-1 (Qin et al., 2012)
27		Rha-(1→2)-Glc		α-OH			sansevierin A (Qin et al., 2012)
28		Rha-(1→4)-Glc		α-OH			disoseptemloside D (Qin et al., 2012)
29		Ara-(1→4)-Glc		β-OH			(25R)-spirost-5-en-3*β*, 7*β*-diol-3-O-*β*-D-arabinofuranosyl-(1→4)-*β*-D-glucopyranoside (Wu et al., 2012a)
30		Ara-(1→4)-[Rha-(1→2)]-Glc		α-OH			parisyunnanoside E (Zhao et al., 2007)
31		Ara-(1→4)-[Rha-(1→2)]-Glc		β-OH			parisyunnanoside D (Zhao et al., 2007)
32		Glc-(1→3)-[Rha-(1→2)]-Glc		β-OH			(25R)-spirost-5-en-3*β*, 7*β*-diol-3-O-*β*-D-glucopyranosyl-(1→3)-[*α*-L-rhamnopyranosyl-(1→2)]-*β*-D-glucopyranoside (Wu et al., 2012a)
33		Rha-Glc-Rha		α-OH			dioseptemloside G (Tao et al., 2020)
34		Rha-(1→2)-Glc-[Rha-(1→4)]-Glc		α-OH			disoseptemloside E (Qin et al., 2012)
		R_1_		R_2_	R_3_		
35	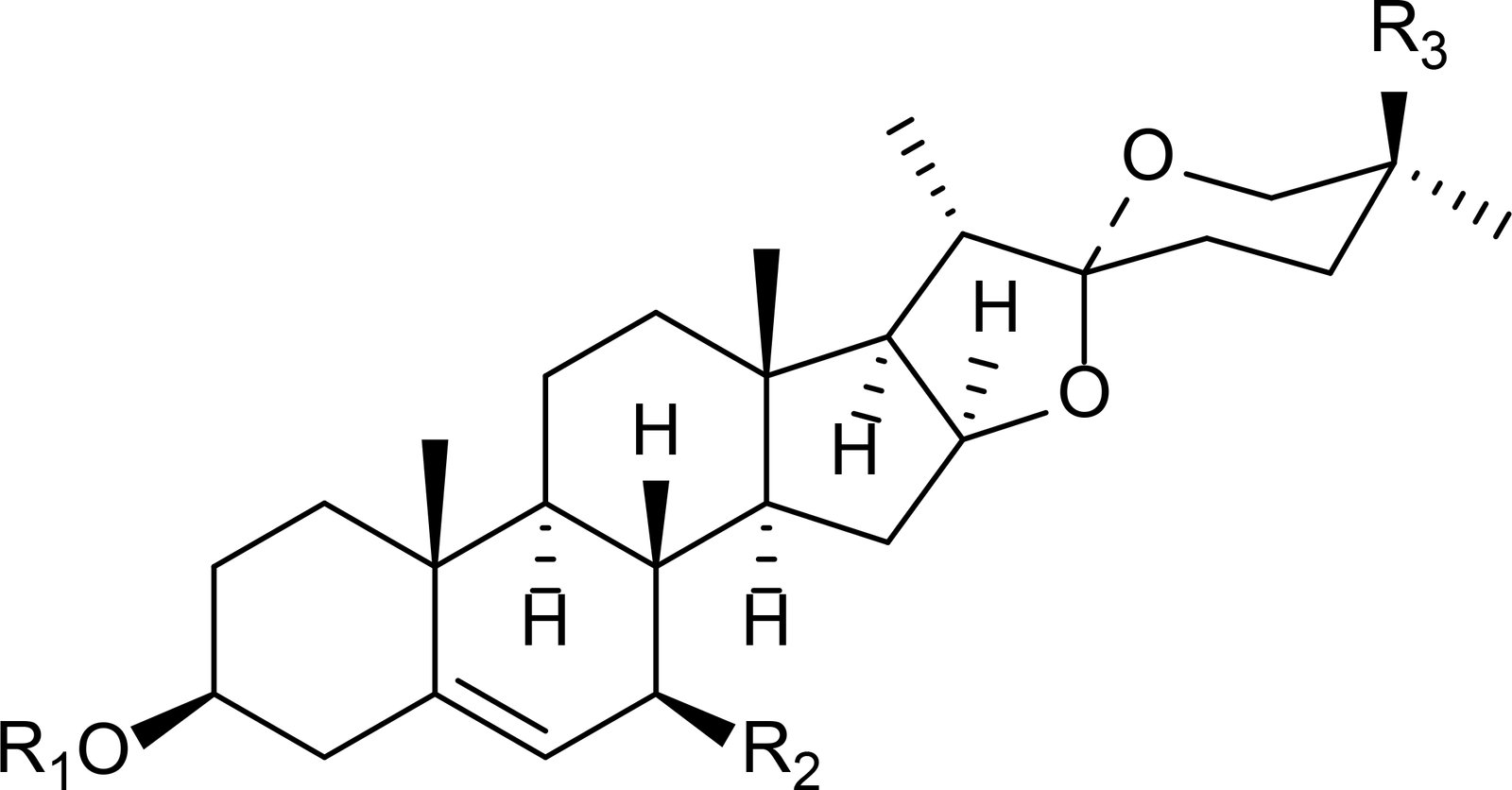	Rha-(1→2)-[Rha-(1→4)]-Glc		OEt	H		chonglouoside SL-16 (Qin et al., 2016)
36	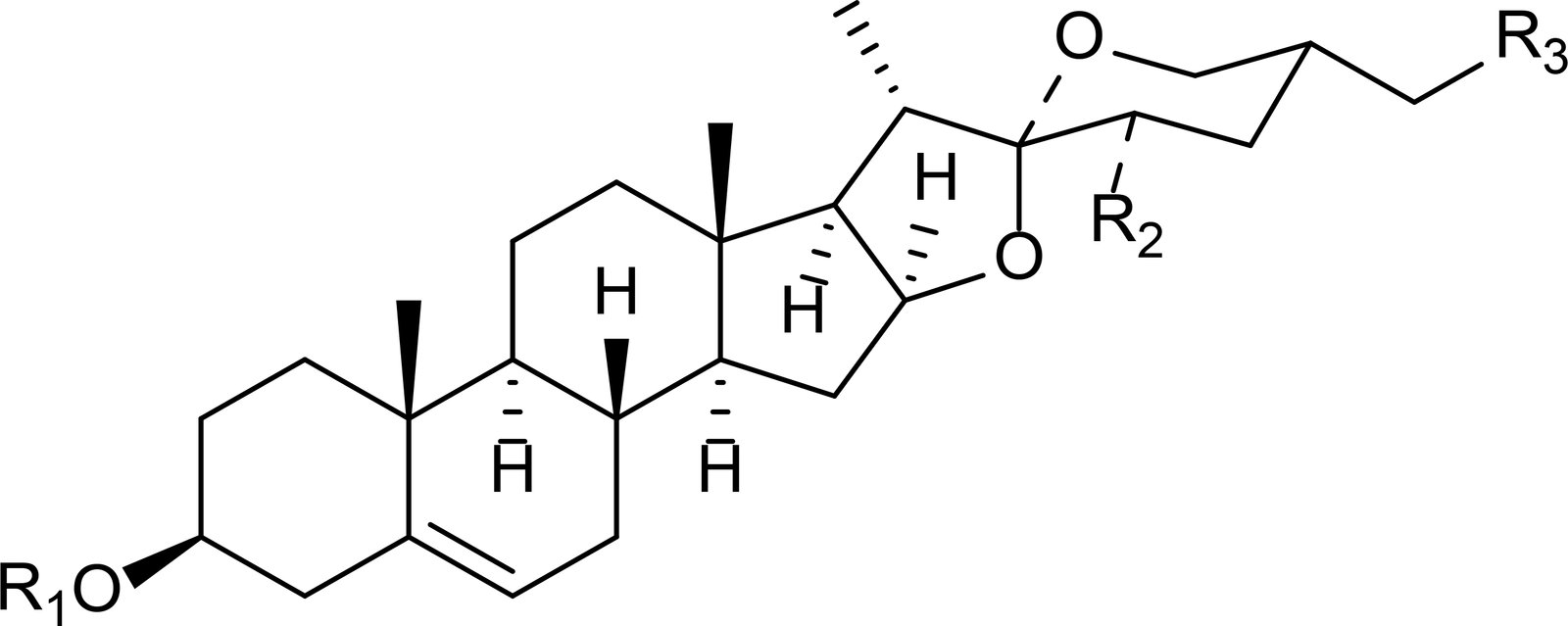	Glc		O-Glc	OH		chonglouoside SL-17 (Qin et al., 2016)
37		Rha-(1→4)-Glc		OH	OH		chonglouoside SL-2 (Qin et al., 2012)
38		Glc-(1→6)-Glc		O-Glc	OH		chonglouoside SL-18 (Qin et al., 2016)
39		Rha-(1→2)-[Rha-(1→4)]-Glc		O-Glc	OH		chonglouoside SL-3 (Qin et al., 2012)
40		Rha-(1→2)-[Rha-(1→4)]-Glc		OH	O-Glc		chonglouoside SL-4 (Qin et al., 2012)
41		Rha-(1→2)-[Rha-(1→4)]-Glc		OH	OH		borassoside B (Qin et al., 2016)
42		Rha-(1→2)-[Ara-(1→4)]-Glc		H	OH		dianchonglouoside B (Wen et al., 2015b)
43		Rha-(1→4)-Rha-(1→4)-[Rha-(1→4)]-Glc		H	OH		(3*β*, 25S)-spirost-5-ene-3, 27-diol-3-O-*α*-L-rhamnopyranosyl-(1→4)-*α*- L-rhamnopyranosyl-(1→4)-[*α*-L-rhamnopyranosyl-(1→2)]-*β*-D-glucopyranoside (Wu et al., 2012a)
		R_1_	R_2_	R_3_	R_4_		
44	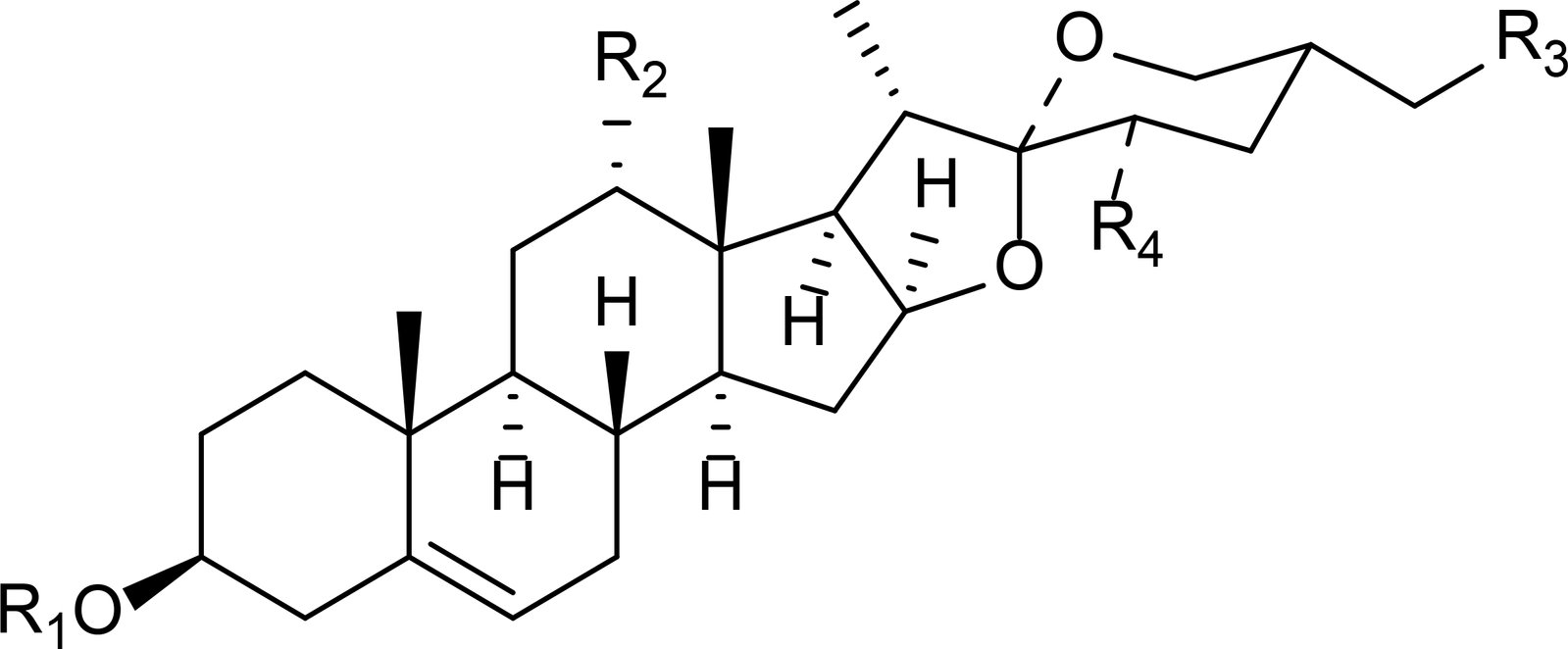	Rha-(1→4)-Glc	OH	OH	OH		Paripolin F (Nie et al., 2024)
45		Rha-(1→2)-[Rha-(1→4)]-Glc	OH	OH	OH		paripolin D (Nie et al., 2024)
46		Rha-(1→4)-Rha-(1→4)-Glc	OH	OH	OH		Paripolin E (Nie et al., 2024)
		R_1_	R_2_	R_3_	R_4_	R_5_	
47	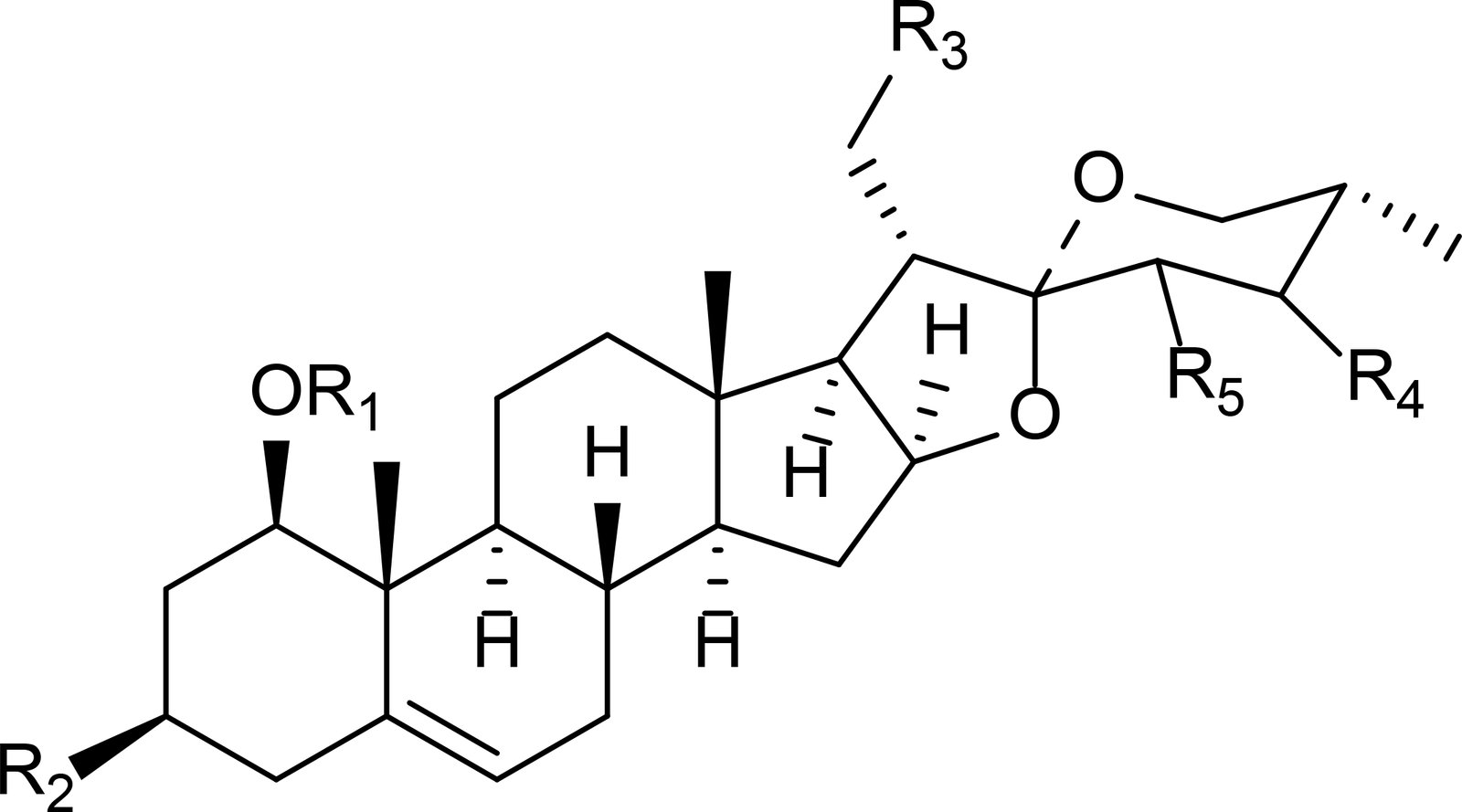	Glc-(1→5)-Glc	OH	OH	OH	OH	paris saponin XI (Wen et al., 2015b)
48	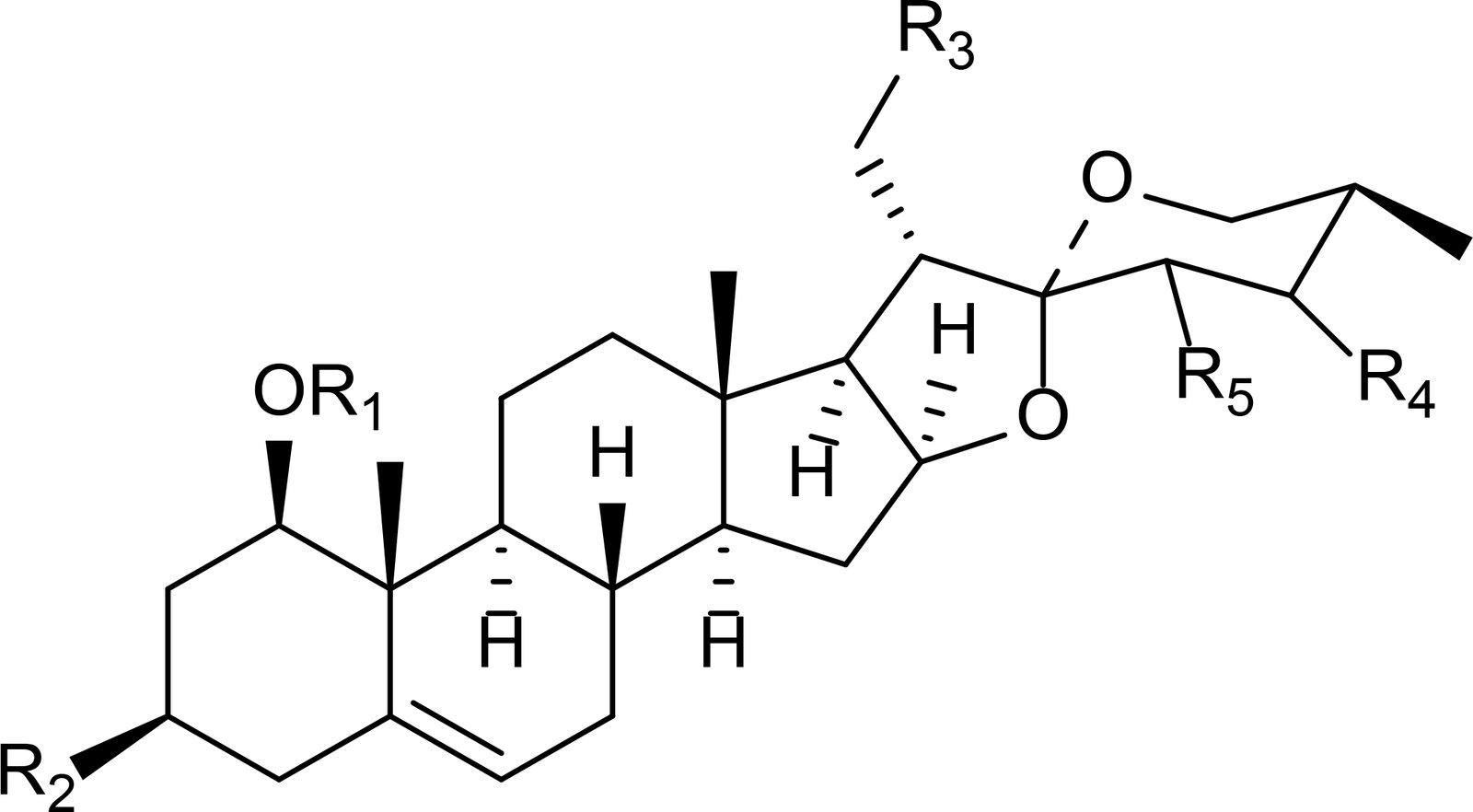	Rha-(1→2)-[Xyl-(1→3)]-Glc	OH	OH	O-Fuc	OH	padelaoside B (Kang et al., 2012)

#### 4.2.2 Protodiosgenin-type and pseudoprotodiosgenin-type saponins

**Figure 5:**
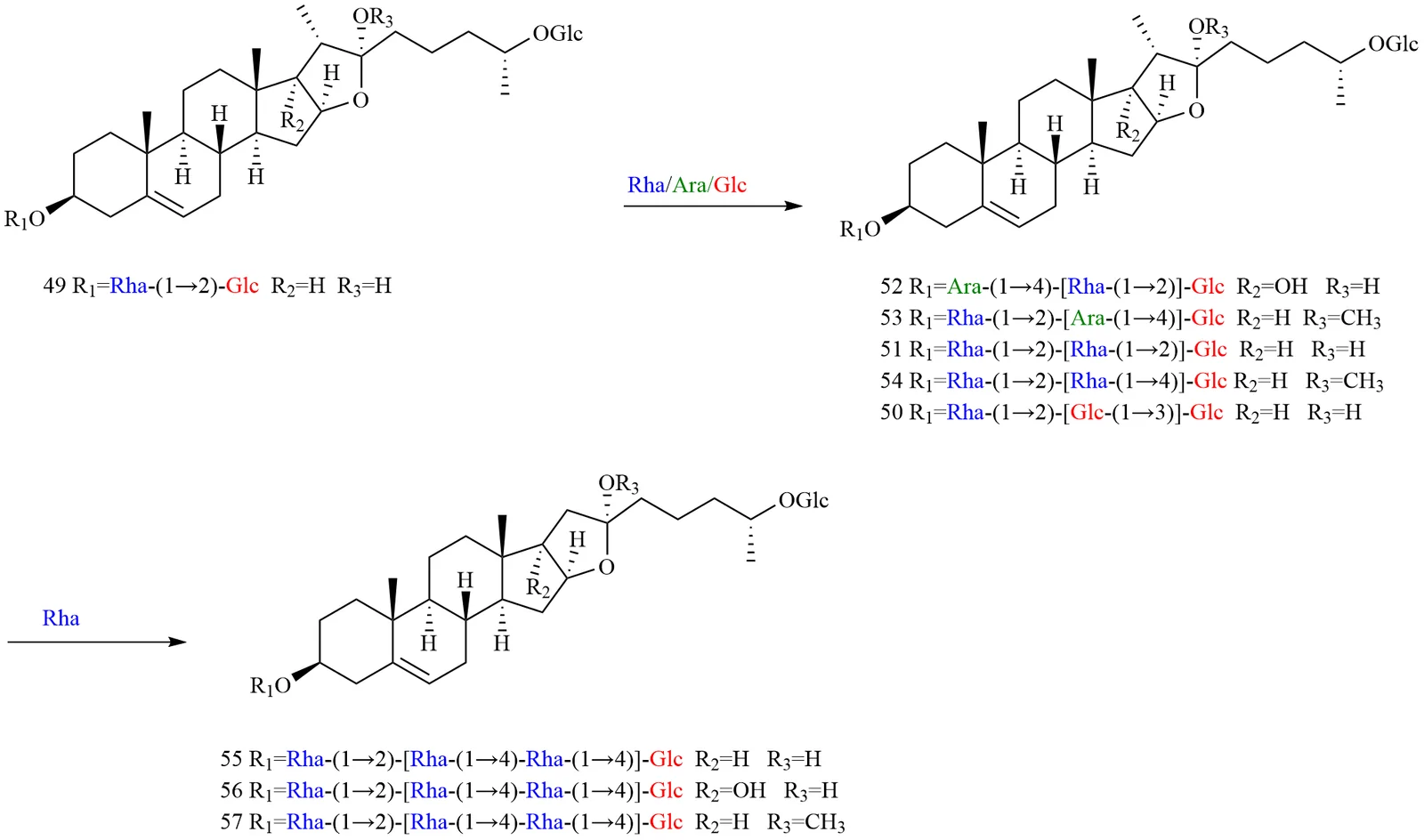
Glycosylation pattern of protodiosgenin-type saponins.

**Figure 6:**
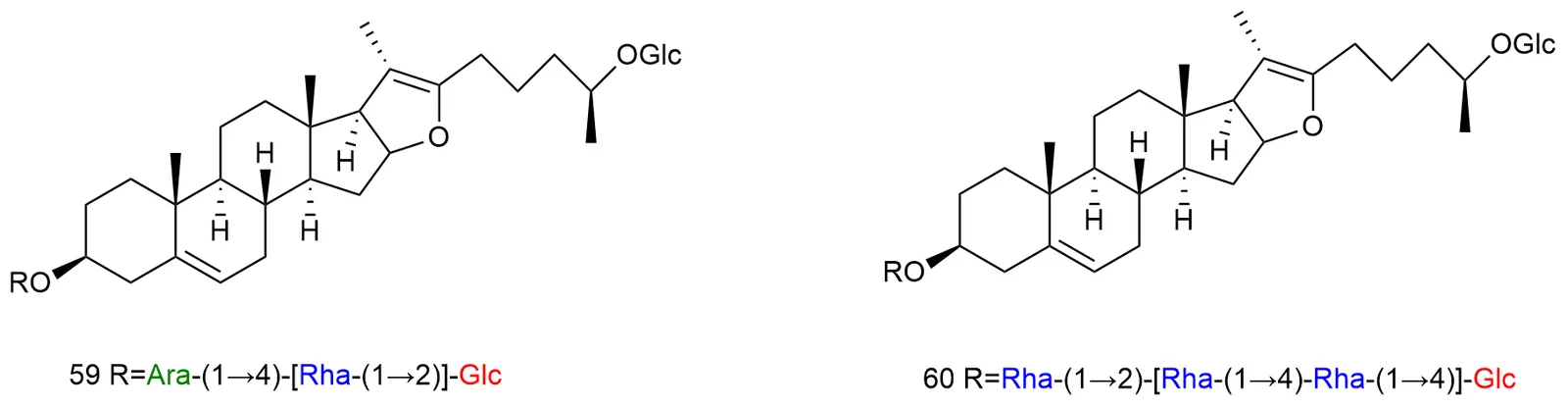
Glycosylation pattern of pseudoprotodiosgenin-type saponins.

A total of 10 protodiosgenin-type saponins (49-58) and two pseudoprotodiosgen-in-type saponins (59-60) have been identified in *P*. *polyphylla*. Structural modifications of protodiosgenin-type saponins mainly occur at positions C-3, C-7, C-17, and C-22. Consistent with diosgenin-type compounds, the primary site of glycosylation is the C-3 position, while the other positions are predominantly substituted with methyl and hydroxyl groups. The innermost sugar residue at C-3 is the same as that in diosgen-in-type saponins. The sugar chains attached to the C-3 position are mostly trisaccharides and tetrasaccharides. Trisaccharide chains are typically formed by further addition of Rha, Ara, or Glc units to the disaccharide backbone Rha-(1→2)-Glc, whereas tetrasaccharide chains are extended by adding additional Rha units to the trisaccharide back-bone Rha-(1→2)-[Rha-(1→4)]-Glc (**Table 2**; **Figure 5**). In the aglycone skeleton of pseudoprotodioscin-type saponins, a double bond exists between the C-20 and C-22 positions. Substitutions are mainly located at the C-3 position, where glycosylation occurs to yield compounds such as parisyunnanoside B and pseudoproto-Pb (**Table 3**; **Figure 6**).

**Table 2:** Protodiosgenin-type saponins from *P*. *polyphylla*.

NO.	Aglycone skeleton	Substituent			Compound name
		R_1_	R_2_	R_3_	
49	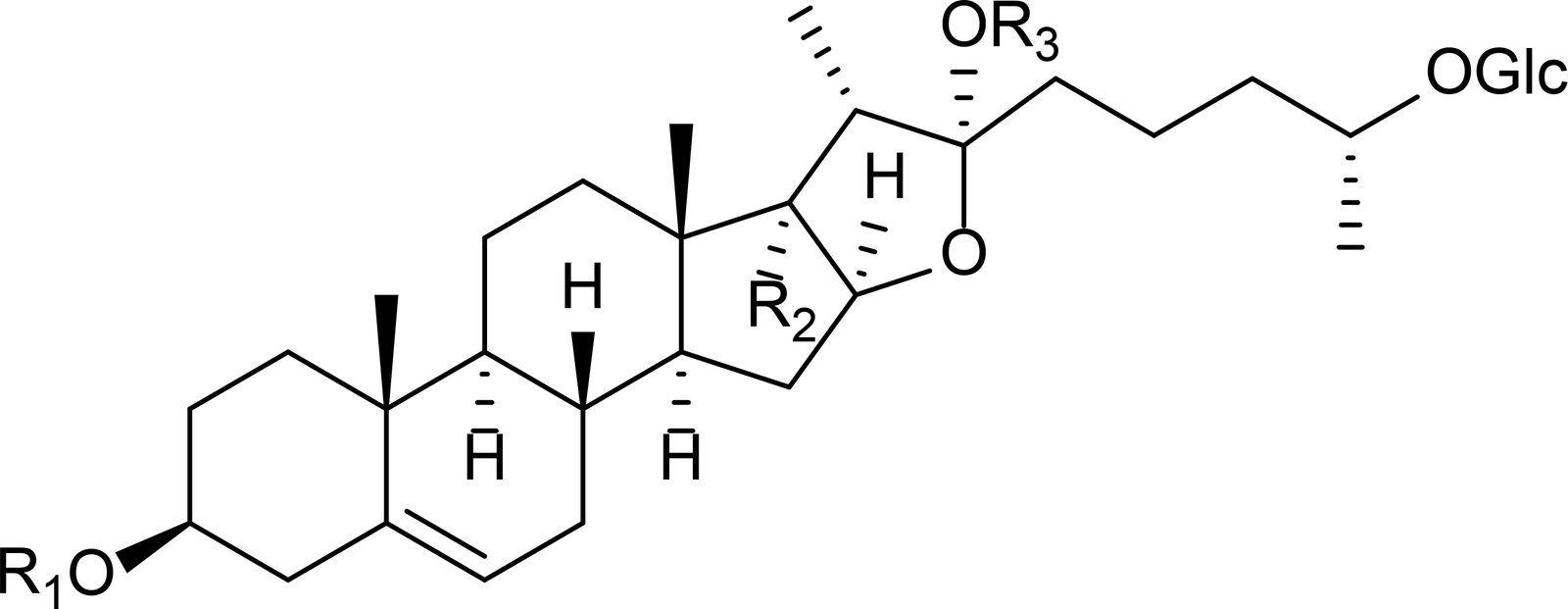	Rha-(1→2)-Glc	H	H	trigofoenoside A (Matsuda et al., 2003)
50		Rha-(1→2)-[Glc-(1→3)]-Glc	H	H	protogracillin (Matsuda et al., 2003)
51		Rha-(1→2)-[Rha-(1→2)]-Glc	H	H	parisaponin I (Matsuda et al., 2003; Qin et al., 2012)
52		Ara-(1→4)-[Rha-(1→2)]-Glc	OH	H	parisyunnanoside A (Zhao et al., 2009)
53		Rha-(1→2)-[Ara-(1→4)]-Glc	H	CH_3_	26-O-*β*-D-glucopyranosyl-(25R)-22-methoxy-furost-5-en-3*β*, 26-diol-3-O-*α*-L-rhamnopyranosyl-(1→2)-[*β*-D-arabinofuranosyl-(1→4)]-*β*-D-glucopyranoside (Miyamura et al., 1982)
54		Rha-(1→2)-[Rha-(1→4)]-Glc	H	CH_3_	26-O-*β*-D-glucopyranosyl-(25R)-22-methoxy-furost-5-en-3*β*, 26-diol- 3-O-*α*-L-rhamnopyranosyl-(1→2)-[*α*-L-rhamnopy-ranosyl (1→4)]-*β*-D-glucopyranoside (Qin et al., 2012)
55		Rha-(1→2)-[Rha-(1→4)-Rha-(1→4)]-Glc	H	H	dichotomin (Zhao et al., 2009)
56		Rha-(1→2)-[Rha-(1→4)-Rha-(1→4)]-Glc	OH	H	saponin Th (Zhao et al., 2009)
57		Rha-(1→2)-[Rha-(1→4)-Rha-(1→4)]-Glc	H	CH_3_	26-O-*β*-D-glucopyranosyl-(25R)-22-methoxy-furost-5-en-3*β*, 26-diol- 3-O-*α*-L-rhamnopyranosyl-(1→2)-[*α*-L-rhamnopy-ranosyl-(1→4)-*α*-L- rhamnopyranosyl (1→4)]-*β*-D-glucopyranoside (Miyamura et al., 1982)
58	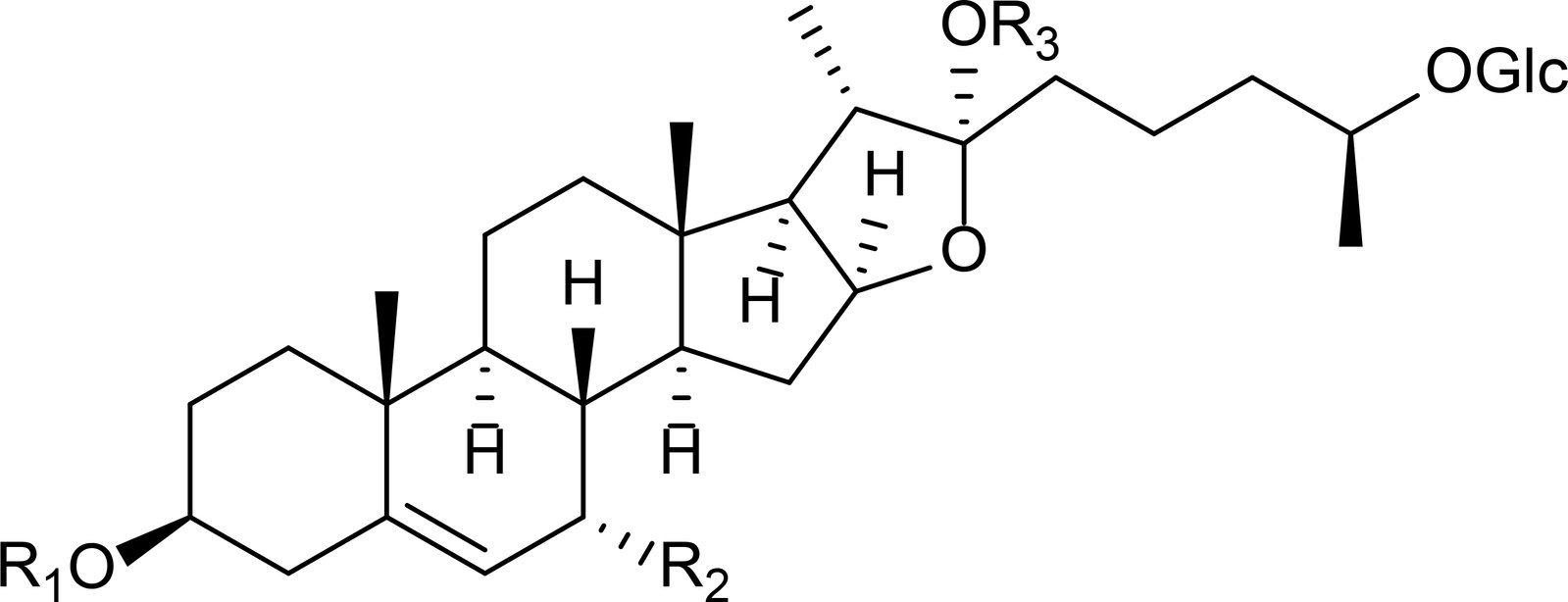	Rha-(1→2)-[Rha-(1→4)]-Glc	OH	H	chonglouoside SL-20 (Qin et al., 2016)

**Table 3:** Pseudoprotodioscin-type saponins from *P*. *polyphylla.*

NO.	Aglycone skeleton	Substituent	Compound name
		R_1_	
59	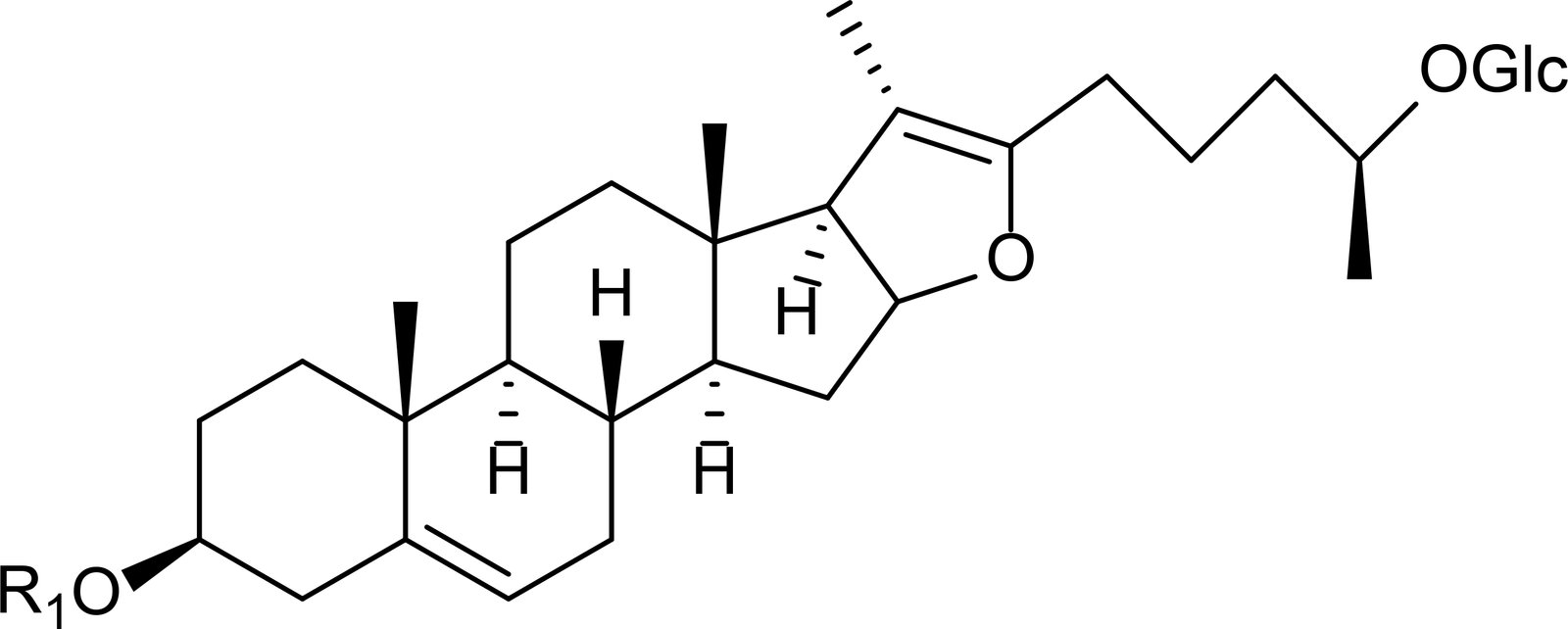	Ara-(1→4)-[Rha-(1→2)]-Glc	parisyunnanoside B (Zhao et al., 2009)
60		Rha-(1→2)-[Rha-(1→4)-Rha-(1→4)]-Glc	pseudoproto-Pb (Zhao et al., 2009)

#### 4.2.3 Pennogenin-type saponins

**Figure 7:**
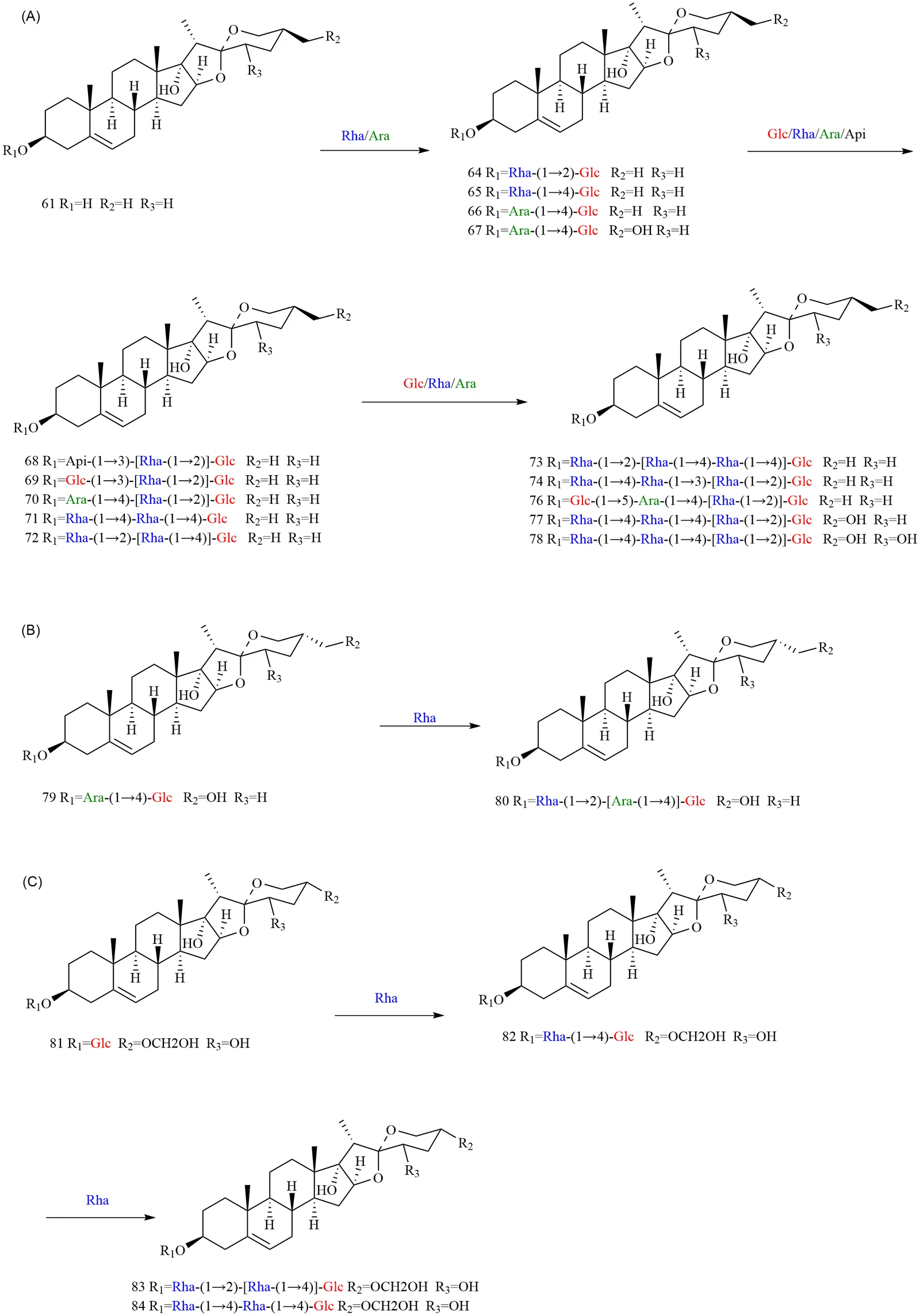
Glycosylation pattern of pennogenin-type saponins. **(A–C)** represent the distinct skeletons formed by different configurations at the C-26 position of the pennogenin-type steroidal saponin aglycone, where **(A)** corresponds to the *β*-configuration, **(B)** to the α-configuration, and **(C)** to a single-bond linkage.

A total of 25 pennogenin-type saponins (61-85) have been identified in *P*. *polyphylla*. The defining structural feature of this class is the hydroxyl substitution at the C-17 position of the aglycone, which represents the most fundamental distinction from diosgenin-type saponins. Structural modifications mainly occur at the C-3, C-23, C-25, and C-27 positions. In addition to glycosylation at the C-3 position, the C-23 position can also undergo hydroxylation and subsequently form a glycosidic linkage with Glc. The glycosidic chains attached at the C-3 position of pennogenin-type saponins are pre-dominantly disaccharide, trisaccharide, and tetrasaccharide moieties. The disaccharide chains mainly include three types: Rha-(1→2)-Glc, Rha-(1→4)-Glc, and Ara-(1→4)-Glc. Trisaccharide chains are primarily formed by the addition of Glc, Rha, Ara, or apiose (Api) to the reducing end of these disaccharide cores, whereas tetrasaccharide chains are further extended or branched based on these structures (**Table 4**; **Figure 7**).

**Table 4:** Pennogenin-type saponins from *P*. *polyphylla*.

NO.	Aglycone skeleton	Substituent			Compound name
		R_1_	R_2_	R_3_	
61	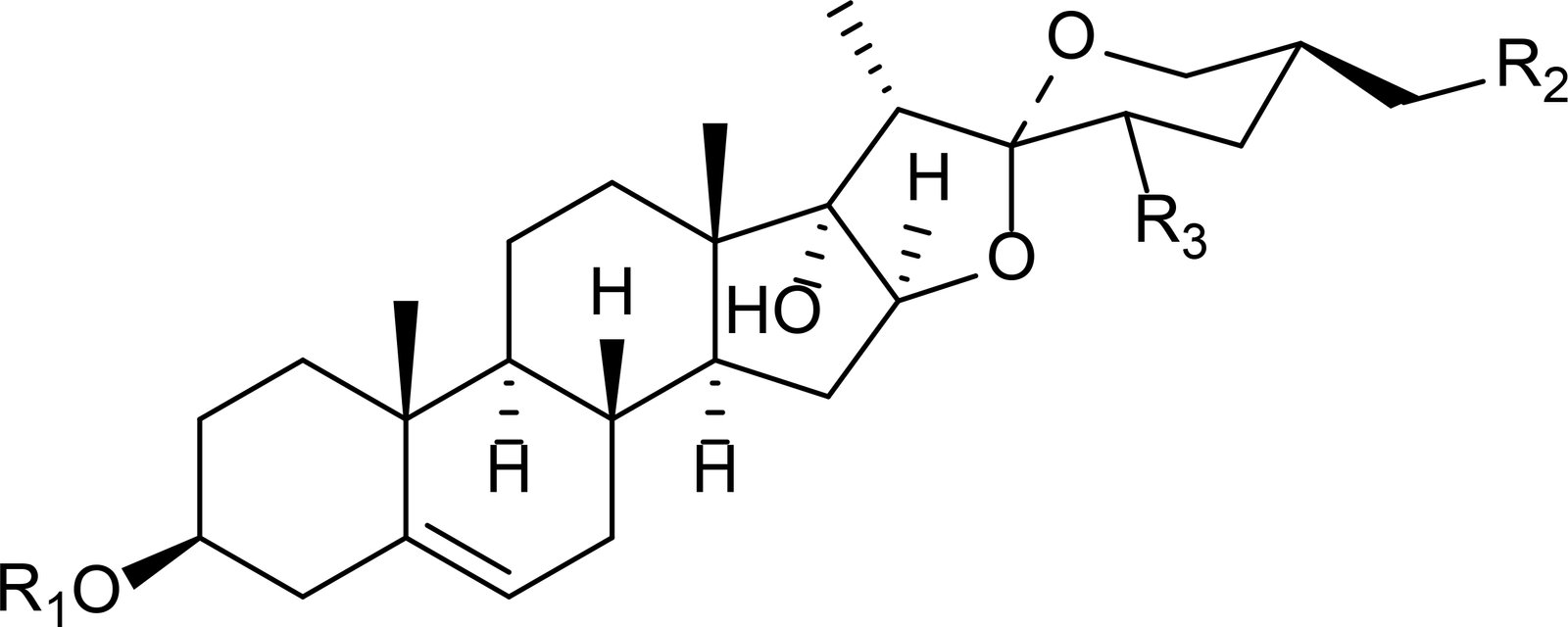	H	H	H	pennogenin (Huang, 1965)
62		H	OH	H	27-ol-pennogenin (Chen and Zhou, 1992; Guan et al., 2019; Wei et al., 2014)
63		OH	OH	OH	27, 23*β*-diol-pennogenin (Chen and Zhou, 1992)
64		Rha-(1→2)-Glc	H	H	polyphyllin VII (Qin et al., 2012)
65		Rha-(1→4)-Glc	H	H	pennogenin-3-O-*α*-L-rhamnopyranosyl-(1→4)-*β*-D-glucopyranoside (Wei et al., 2014)
66		Ara-(1→4)-Glc	H	H	pennogenin-3-O-*α*-L-arabinofuranosyl-(1→4)-*β*-D-glucopyranoside (Wei et al., 2014; Wu et al., 2012a; Zhao et al., 2009)
67		Ara-(1→4)-Glc	OH	H	27-hydroxypennogenin 3-O-*α*-L-arabinofuranosyl-(1→4)-*β*-D-glucopyranoside (Guan et al., 2019; Wei et al., 2014; Wu et al., 2012a)
68		Api-(1→3)-[Rha-(1→2)]-Glc	H	H	pennogenin-3-O-*α*-L-rhamnopyranosyl-(1→2)-[*β*-D-apiofuranosyl-(1→3)]-*β*-D-glucopyranoside (Wu et al., 2012a)
69		Glc-(1→3)-[Rha-(1→2)]-Glc	H	H	pennogenin-3-O-*α*-L-rhamnopyranosyl-(1→2)-[*β*-D-glucopyranosyl-(1→3)]-*β*-D-glucopyranoside (Chen and Zhou, 1981)
70		Ara-(1→4)-[Rha-(1→2)]-Glc	H	H	paris saponin H(polyphyllin H) (Miyamura et al., 1982)
71		Rha-(1→4)-Rha-(1→4)-Glc	H	H	pennogenin-3-O-*α*-L-rhamnopyranosyl-(1→4)-*α*-L-rhamnopyranosyl-(1→4)-*β*-D-glucopyranoside (Qin et al., 2012)
72		Rha-(1→2)-[Rha-(1→4)]-Glc	H	H	polyphyllin B (Chen et al., 1990b; Zhao et al., 2009)
73		Rha-(1→2)-[Rha-(1→4)-Rha-(1→4)]-Glc	H	H	polyphyllin VII (Chen et al., 1990a; Miyamura et al., 1982)
74		Rha-(1→4)-Rha-(1→3)-[Rha-(1→2)]-Glc	H	H	pennogenin-3-O-*α*-L-rhamnopyranosyl-(1→4)-*α*-L-rhamnopyranosyl-(1→3)-[*α*-L-rhamnopyranosyl-(1→2)]-*β*-D-glucopyranosid (Guan et al., 2019; Wei et al., 2014)
75		Rha-(1→2)-[Xyl-(1→5)-Ara-(1→4)]-Glc	H	H	pennogenin-3-O-*β*-D-xylopyranosyl-(1→5)-*α*-L-arabinofuranosyl-(1→4)-[*α*-L-rhamnopyranosyl-(1→2)]-*β*-D-glucopy-ranosid (Wu et al., 2012a)
76		Glc-(1→5)-Ara-(1→4)-[Rha-(1→2)]-Glc	H	H	pennogenin-3-O-*β*-D-glucopyranosyl-(1→5)-*α*-L-arabinofuranosyl-(1→4)-[rhamnopyranosyl-(1→2)]-*β*-D-glucopyranoside (Wu et al., 2012a)
77		Rha-(1→4)-Rha-(1→4)-[Rha-(1→2)]-Glc	OH	H	Polyphylloside III (Chen and Zhou, 1992; Tao et al., 2020; Wei et al., 2014)
78		Rha-(1→4)-Rha-(1→4)-[Rha-(1→2)]-Glc	OH	OH	Polyphylloside IV (Chen and Zhou, 1995)
79	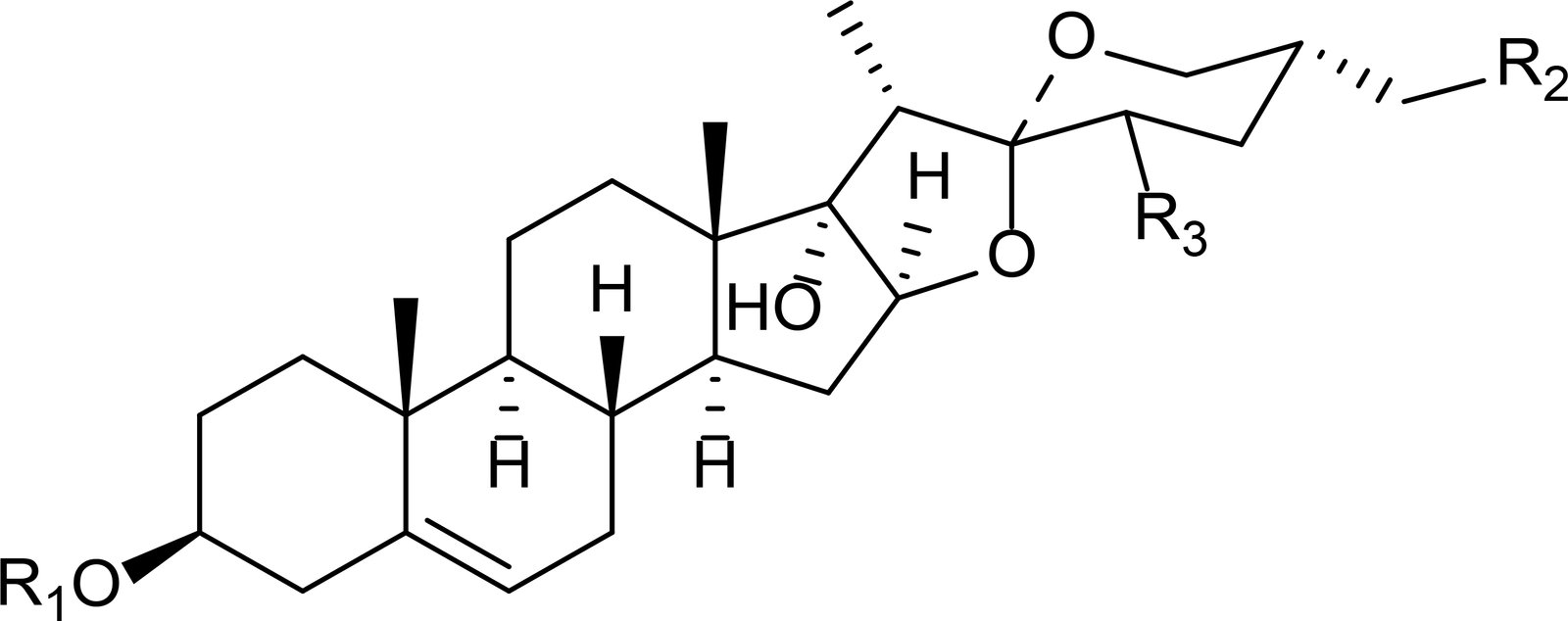	Ara-(1→4)-Glc	OH	H	(3*β*, 17*α*, 25S)-spirost-5-ene-3, 17, 27-triol-3-O-*β*-D-arabinofuranosyl-(1→4)-*β*-D-glucopyranosid (Wu et al., 2012a)
80		Rha-(1→2)-[Ara-(1→4)]-Glc	OH	H	dianchonglouoside A (Wen et al., 2015b)
81	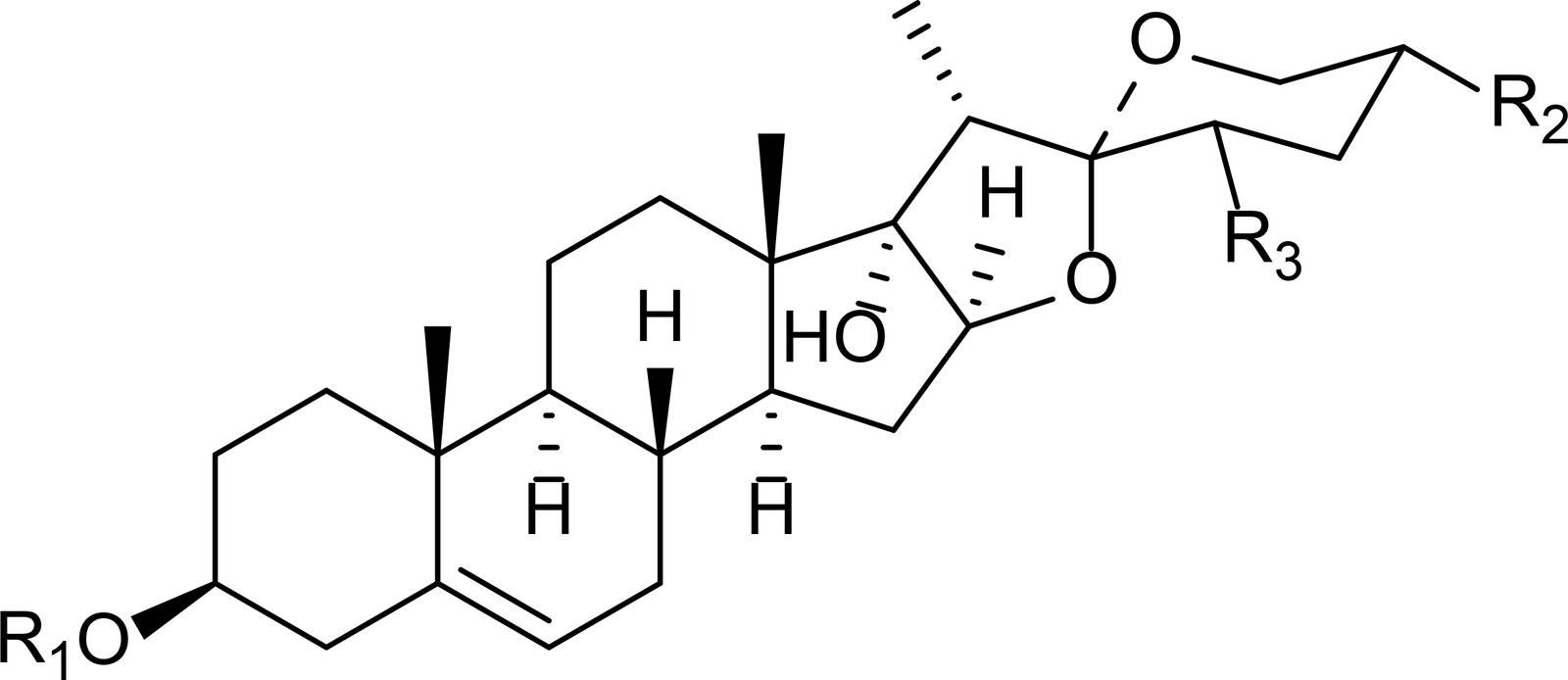	Glc	OCH_2_OH	OH	Paripolin G (Nie et al., 2024)
82		Rha-(1→4)-Glc	OCH_2_OH	OH	(23S, 25S)-17*α*, 23, 27-trihydroxyspirost-5-en-3*β*-yl-O-*α*-L-rhamnopyranosyl-(1→4)-*β*-D-glucopyranoside (Nie et al., 2024)
83		Rha-(1→2)-[Rha-(1→4)]-Glc	OCH_2_OH	OH	(23S, 25S)-5-en-spirost-3*β*, 17*α*, 23*α*, 27-tetraol 3-O-*α*-L-rhamnopyranosyl-(1→4)-*α*-L-rhamnopyranosy1-(1 → 2)-*β*-D-glu­copyranoside (Nie et al., 2024)
84		Rha-(1→4)-Rha-(1→4)-Glc	OCH_2_OH	OH	(23S, 25S)-17*α*, 23, 27-tri­hydroxyspirost-5-en-3*β*-yl-O-*α*-L-rhamnopyranosyl-(1→4)-*β*-D-gluco­pyranoside (Nie et al., 2024)
85		H	OCH_2_OH	O-Glc	Paripolin H (Nie et al., 2024)

#### 4.2.4 Nuatigenin-type and isonuatigenin-type saponins

**Figure 8:**
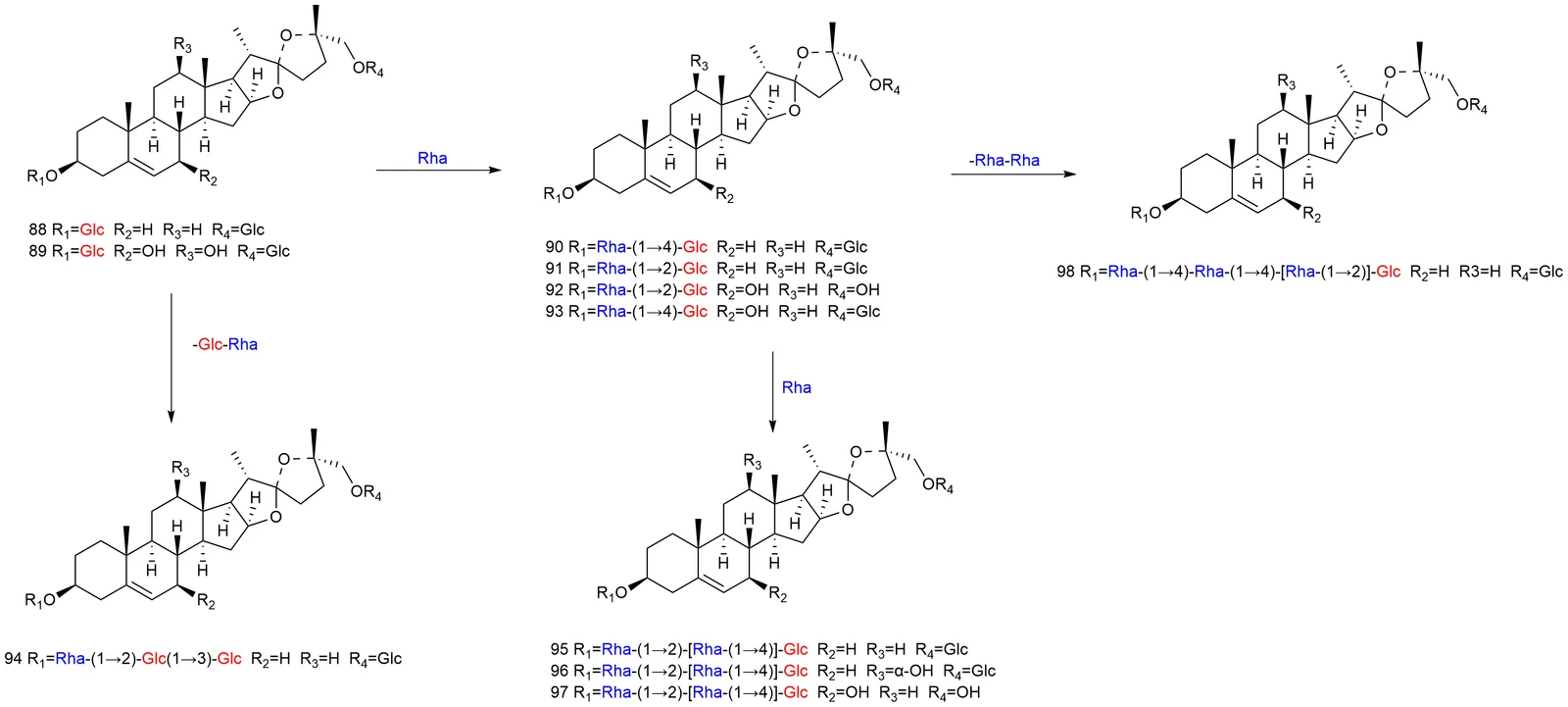
Glycosylation pattern of nuatigenin-type saponins.

**Figure 9:**
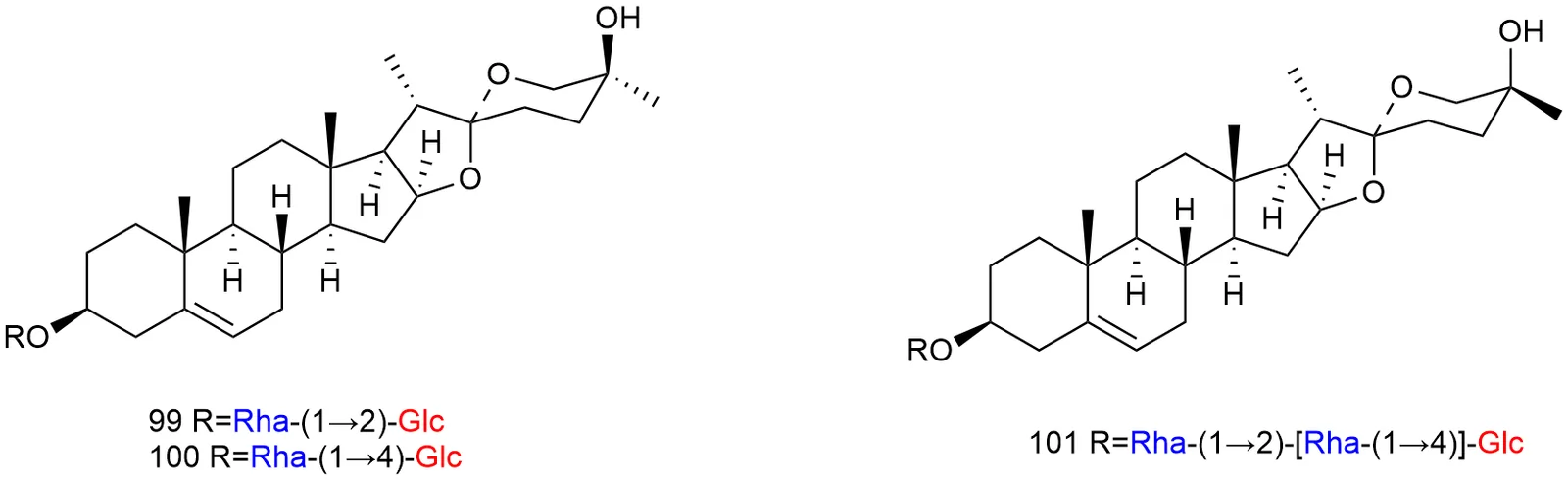
Glycosylation pattern of isonuatigenin-type saponins.

A total of 13 nuatigenin-type saponins (86-98) and 3 isonuatigenin-type saponins (99-101) have been identified in *P*. *polyphylla*. The distinction between these two classes lies in the structural configuration of the F-ring: the F-ring of nuatigenin-type saponins is a furan ring, whereas the F-ring of isonuatigenin-type saponins is also a pyran ring. Structural modifications of nuatigenin-type saponins are mainly concentrated at the C-3, C-7, C-12, and C-26 positions. In addition to glycosylation at the C-3 position, glycosylation can also occur at C-26 through the formation of a glycosidic linkage with Glc. The glycosidic chains attached at C-3 are predominantly monosaccharide, disaccharide, and trisaccharide moieties. The monosaccharide chain consists of a single Glc unit. Disaccharide chains are mainly formed by introducing Rha at the C-2 or C-4 position of Glc. Trisaccharide chains may form branched structures on the Rha-(1→4)-Glc core, yielding the Rha-(1→2)-[Rha-(1→4)]-Glc unit, or adopt a linear structure in which Glc and Rha are attached to a single Glc unit to form Rha-(1→2)-Glc (1→3)-Glc, as exemplified by chonglouoside SL-12. Tetrasaccharide chains are formed by further extension of the disaccharide core Rha-(1→2)-Glc through the addition of two Rha units, resulting in the structure Rha-(1→4)-Rha-(1→4)-[Rha-(1→2)]-Glc (**Table 5**; **Figure 8**). For isonuatigenin-type saponins, the glycosylation site of the aglycone is likewise located at the C-3 position, while the C-27 position is substituted by a hydroxyl group (**Table 6**; **Figure 9**).

**Table 5:** Nuatigenin-type from *P*. *polyphylla*.

NO.	Aglycone skeleton	Substituent				Compound name
		R_1_	R_2_	R_3_	R_4_	
86	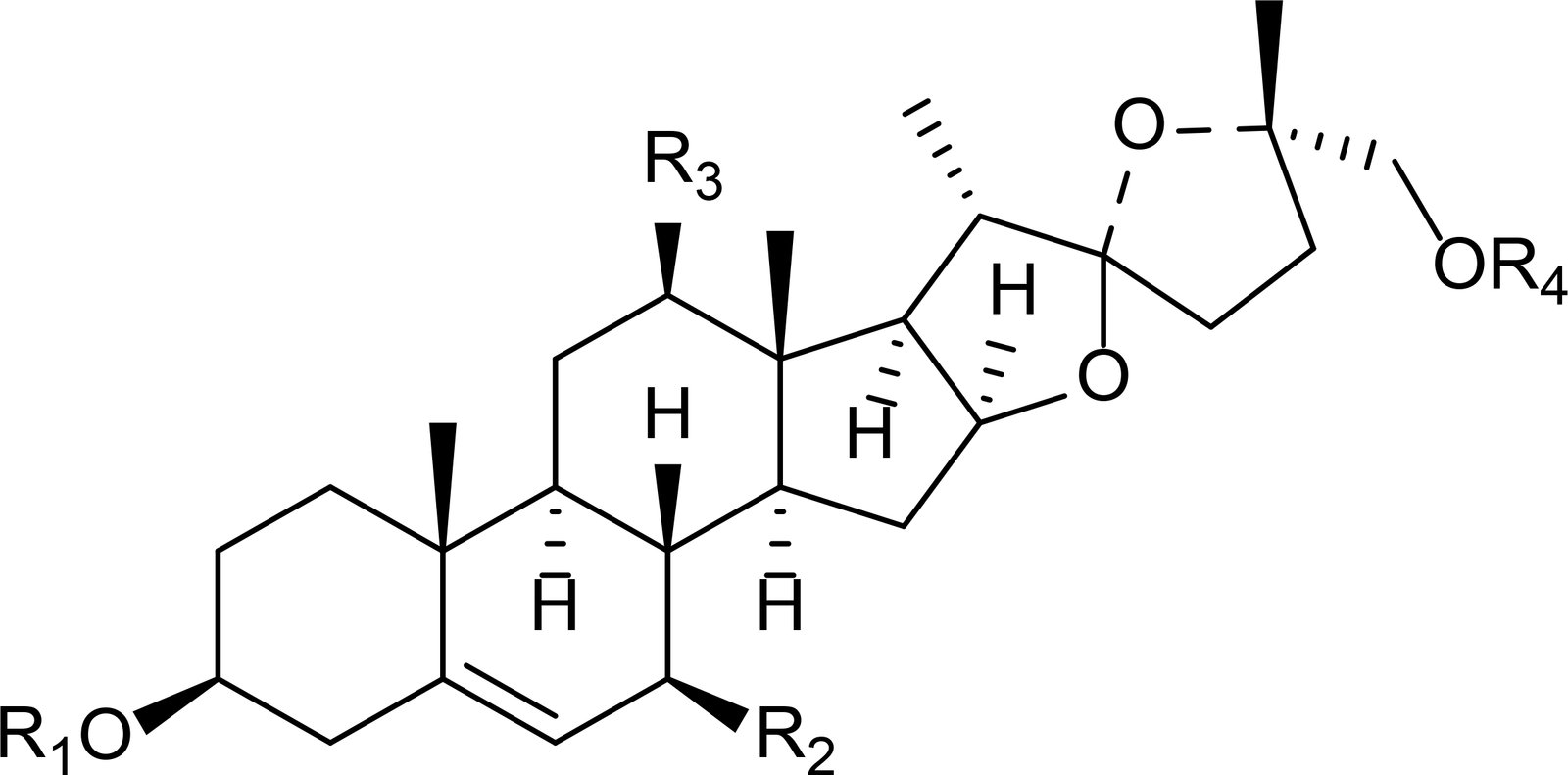	H	H	H	Glc	chonglouoside SL-9 (Qin et al., 2016; Tao et al., 2020)
87		OH	OH	OH	Glc	chonglouoside SL-10 (Qin et al., 2016)
88		Glc	H	H	Glc	chonglouoside SL-11 (Qin et al., 2016)
89		Glc	OH	OH	Glc	chonglouoside SL-13 (Qin et al., 2016)
90		Rha-(1→4)-Glc	H	H	Glc	26-O-*β*-D-glucopyranosyl-nuatigenin-3-O-*α*-L-rhamnopyranosyl-(1→4)-*β*-D-glucopyranoside (Qin et al., 2016)
91		Rha-(1→2)-Glc	H	H	Glc	26-O-*β*-D-glucopyranosyl-nuatigenin-3-O-*α*-L-rhamnopyranosyl-(1→2)-*β*-D-glucopyranoside (Qin et al., 2016)
92		Rha-(1→2)-Glc	OH	H	OH	nuatigenin-3-O-*α*-L-rhamnopyranosyl-(1→2)-*β*-D-glucopyranoside (Qin et al., 2016)
93		Rha-(1→4)-Glc	OH	H	Glc	abutiloside L (Qin et al., 2016)
94		Rha-(1→2)-[Glc-(1→3)]-Glc	H	H	Glc	chonglouoside SL-12 (Qin et al., 2016)
95		Rha-(1→2)-[Rha-(1→4)]-Glc	H	H	Glc	26-O-*β*-D-glucopyranosyl-nuatigenin-3-O-*α*-L-rhamnopyranosyl-(1→2)-[*α*-L-rhamnopyranosyl-(1→4)]-*β*-D-glucopy-ranoside (Chen et al., 1995)
96		Rha-(1→2)-[Rha-(1→4)]-Glc	H	α-OH	Glc	26-O-*β*-D-glucopyranosyl 12*α*-hydroxynuatigenin 3-O-*α*-L-rhamnopyranosyl-(1→2)-[*α*-L-rhamnopyranosyl-(1→4)]-*β*-D-glucopyronoside (Nie et al., 2024)
97		Rha-(1→2)-[Rha-(1→4)]-Glc	OH	H	OH	chonglouoside SL-15 (Qin et al., 2016)
98		Rha-(1→4)-Rha-(1→4)-[Rha-(1→2)]-Glc	H	H	Glc	chonglouoside SL-14 (Qin et al., 2016)

**Table 6:** Isonuatigenin-type from *P*. *polyphylla*.

NO.	Aglycone skeleton	Substituent	Compound name
		R_1_	
99	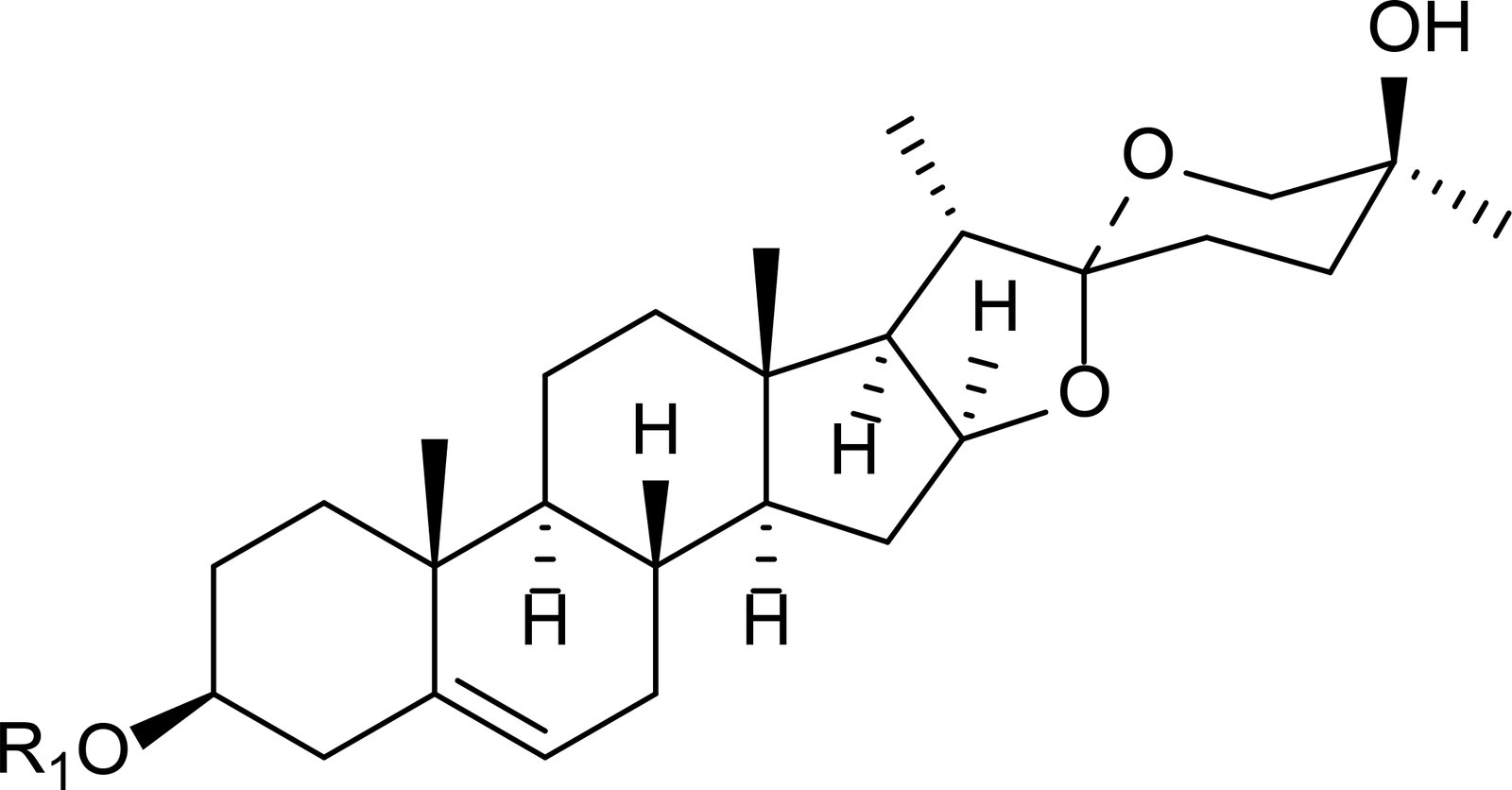	Rha-(1→2)-Glc	(25S)-spirost-5-en-3*β*, 25-diol-3-O-*α*-L-rhamnopyranosyl-(1→2)-*β*-D-glucopyranoside (Qin et al., 2012)
100		Rha-(1→4)-Glc	trigofoenoside A (Chen et al., 1990b)
101	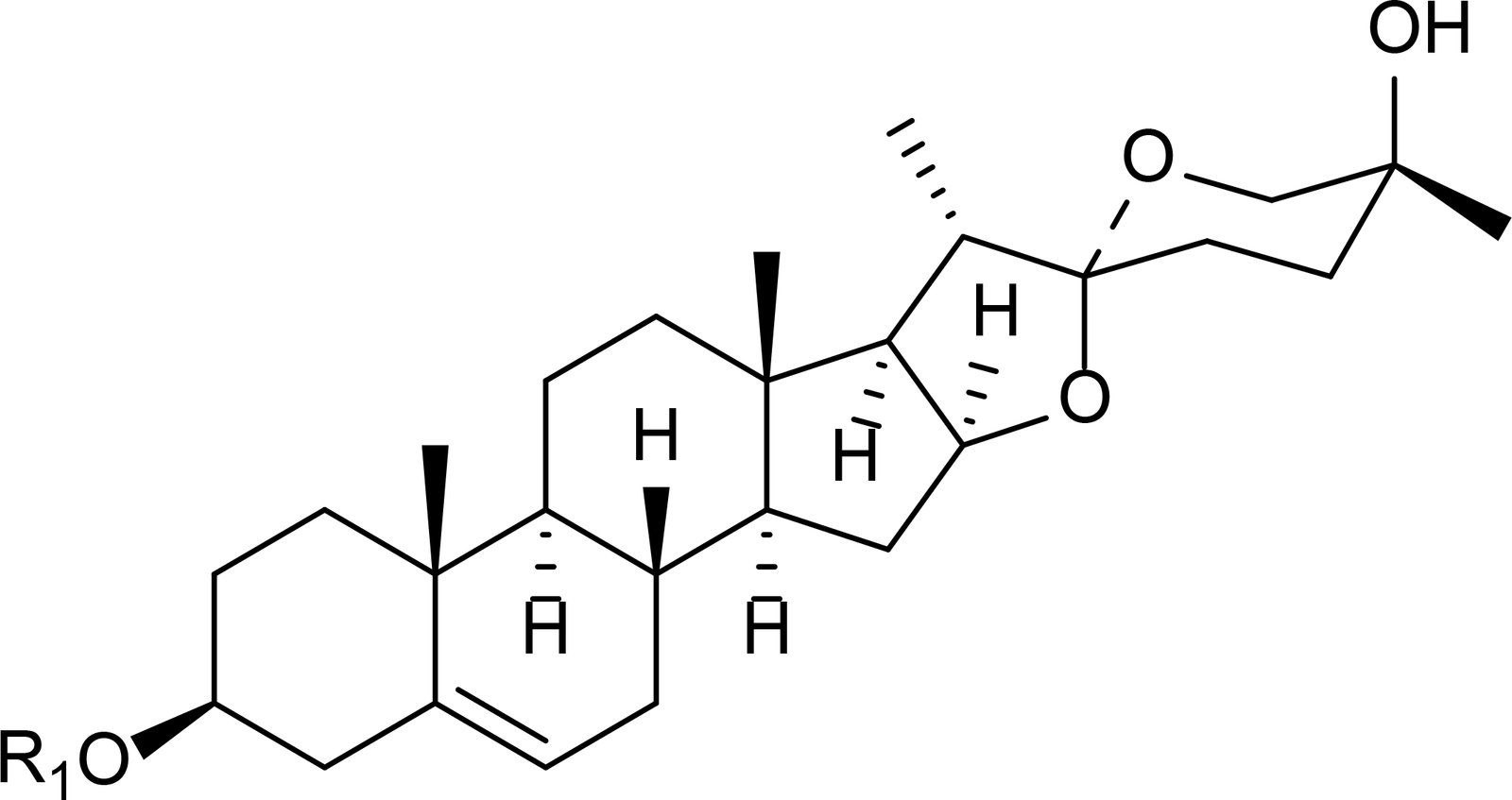	Rha-(1→2)-[Rha-(1→4)]-Glc	25S-isonuatigenin-3-O-*α*-L-rhamnopyranosyl-(1→2)-[*α*-L-rhamnopyranosyl-(1→4)]-*β*-D-glucopyranoside (Chen et al., 1995)

#### 4.2.5 Other-type saponins

A total of 21 nuatigenin-type saponins (102-122) have been identified in *P*. *polyphylla*. In addition to the typical substitutions of hydroxyl, carbonyl, and glycosyl groups on the steroidal aglycone, some of these compounds exhibit distinctive structural features, mainly including oxygen-bridged (114) and peroxide-bridged structures (115-118) (**Table 7**).

**Table 7:** Other-type from *P*. *polyphylla*.

NO.	Aglycone skeleton	Substituent			Compound name
		R_1_			
102	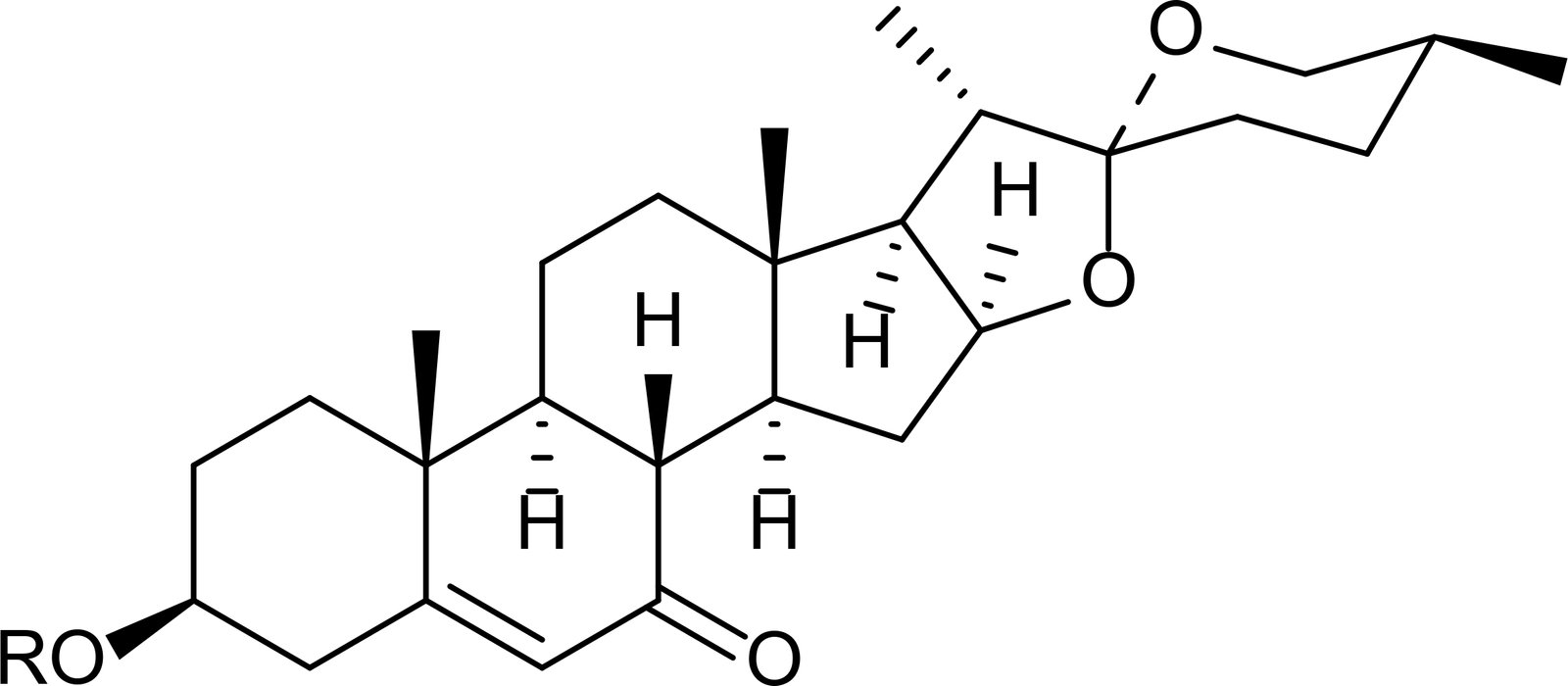	Rha-(1→2)-[Rha-(1→4)-Rha-(1→4)]-Glc			chonglouoside SL-5 (Qin et al., 2012; Tao et al., 2020)
103		Rha-(1→2)-Glc			(3*β*, 25R) -3-ol-spirost-5-en-7-one-3-O-*α*-L-rhamnopyranosyl-(1→2)-*β*-D-glucopyranoside (Wu et al., 2012a)
104	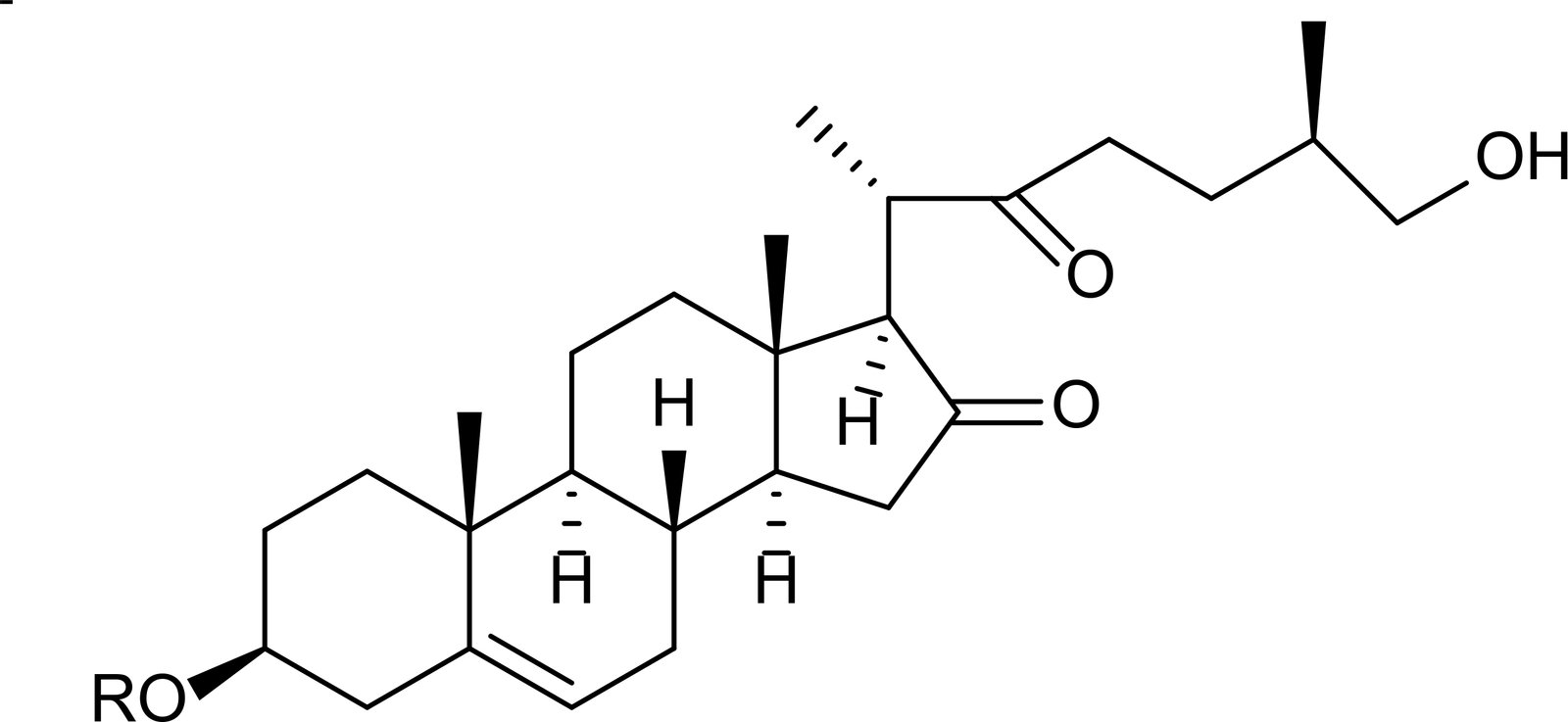	Rha-(1→4)-Rha-(1→4)-[Rha-(1→2)]-Glc			chonglouoside SL-19 (Qin et al., 2016)
105	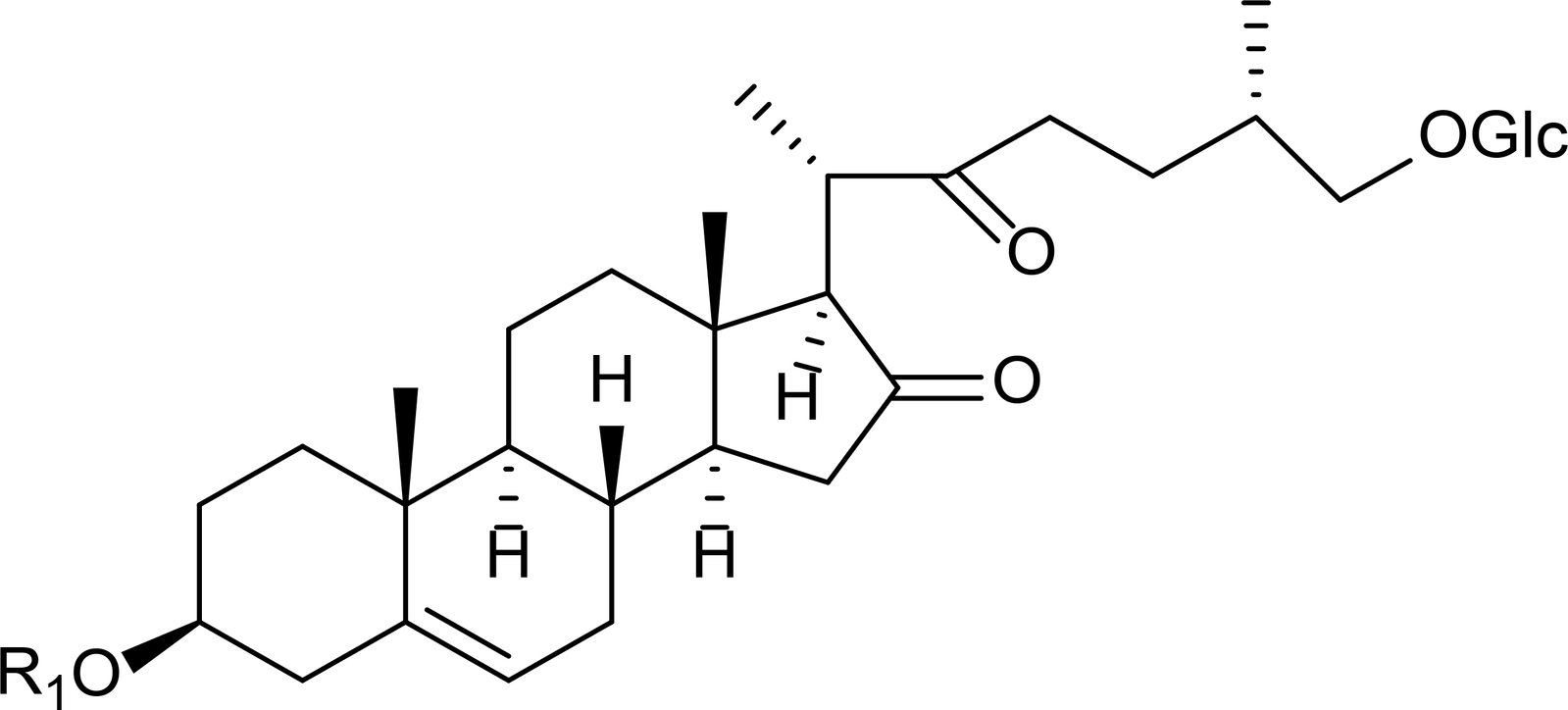	Rha-(1→2)-[Rha-(1→4)]-Glc			3-O-*β*-chacotriosyl kryptogenin 26-O-*β*-D-glucopyranoside (Nie et al., 2024)
106	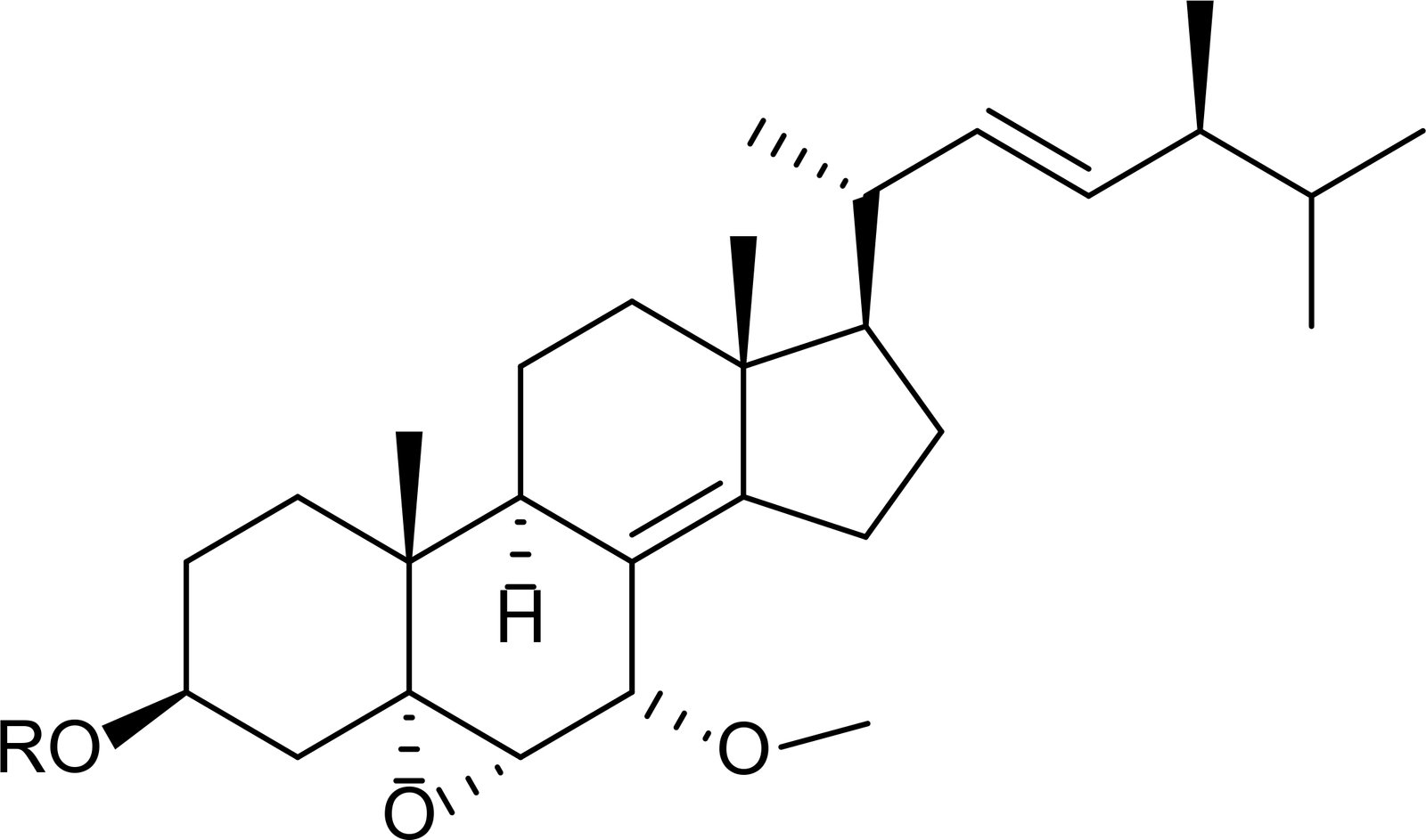	H			22E-7*α*-methoxy-5*α*, 6*α*-epoxyergosta-8(14), 22-dien-3*β*-ol (Jiang et al., 2021)
107	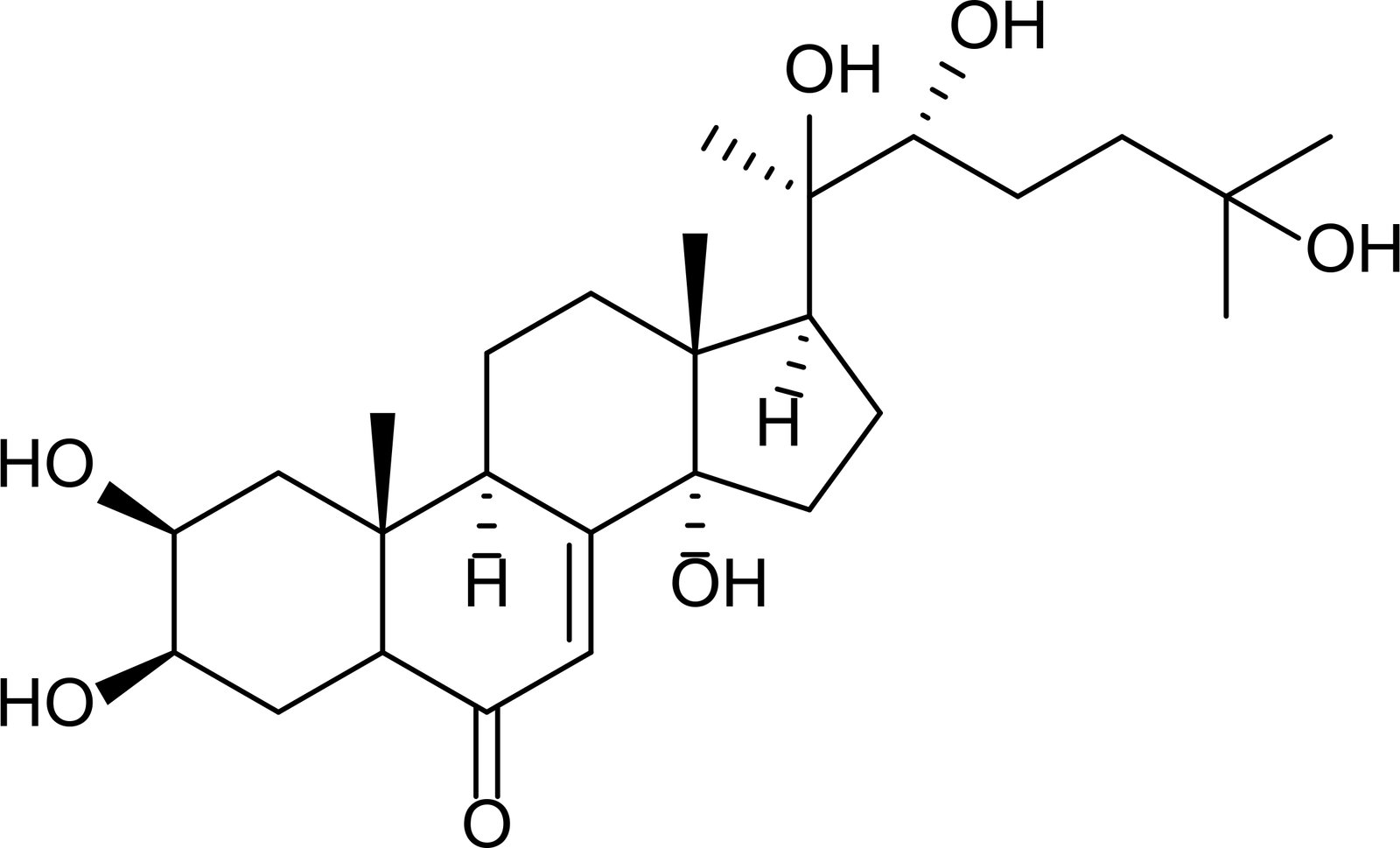	–			20*β*-hydroxyecdysone (Nie et al., 2024)
108	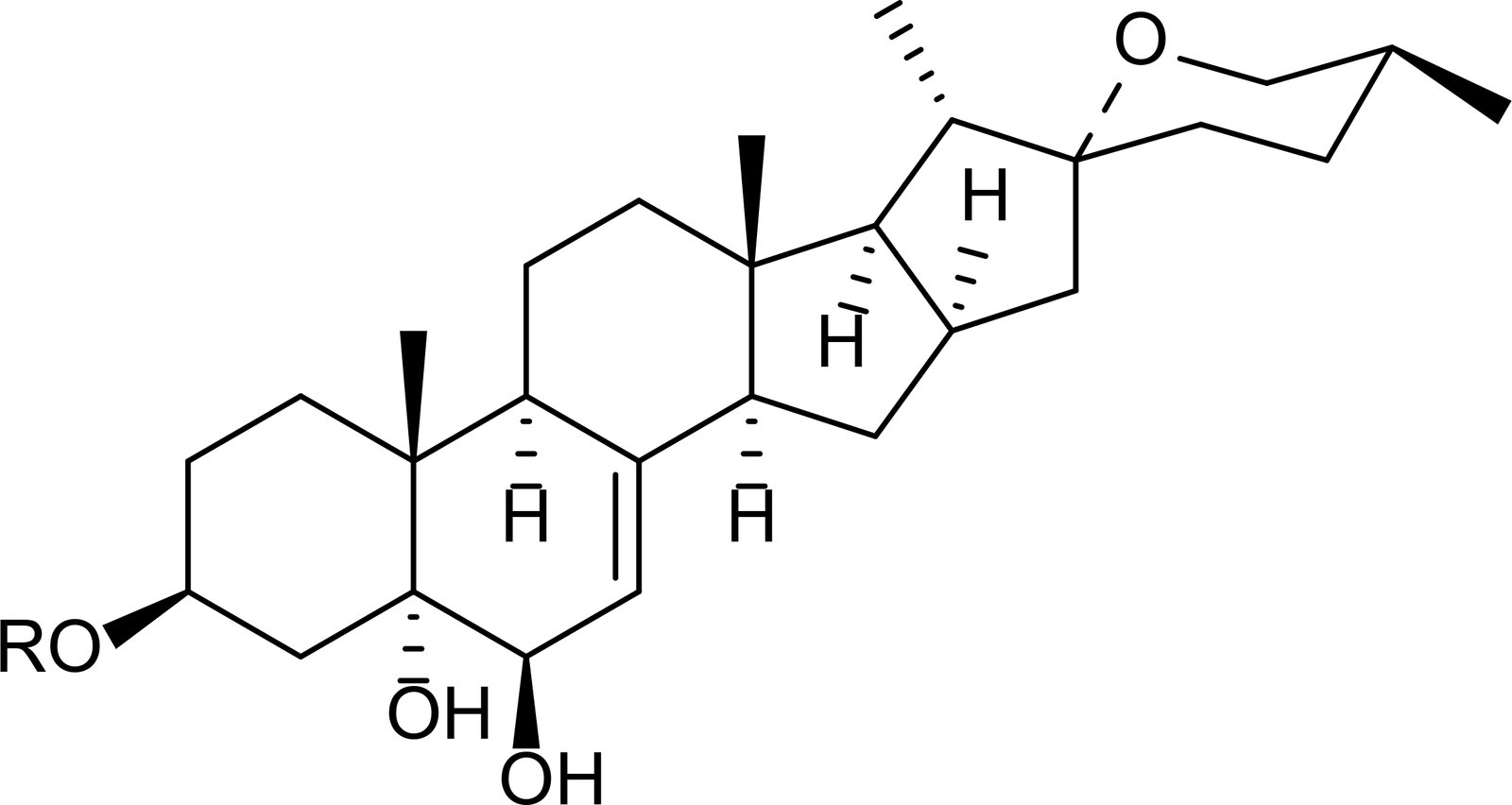	Glc-(1→3)-[Rha-(1→2)-Glc]			(25R)-spirost-7(8)-ene-3*β*, 6*β*-diol-3-O-*β*-D-glucopyranosyl-(1→3)-[*α*-L-rhamnopyranosyl-(1→2)]-*β*-D-glucopyranoside (Zhao et al., 2009)
109	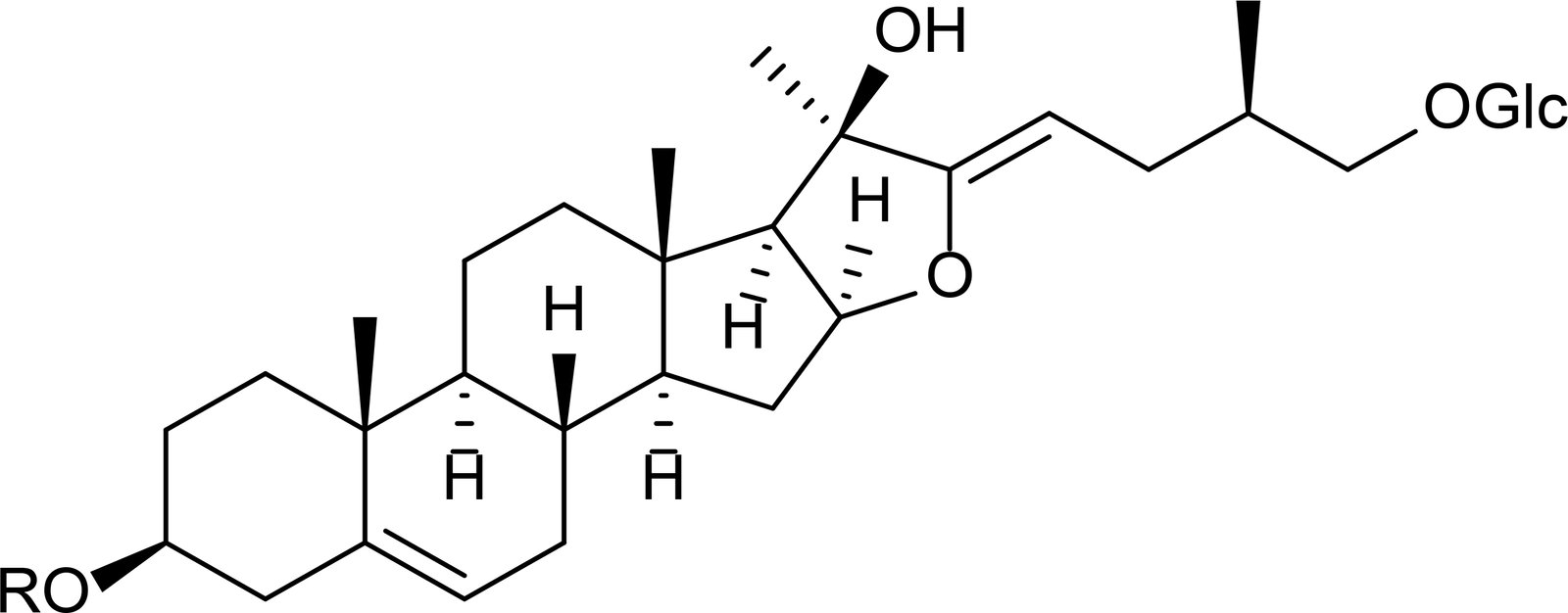	Rha-(1→2)-[Rha-(1→4)]-Glc			26-O-*β*-D-glucopyranosyl-(25R)-5, 22-diene-furost-5-en-3*β*, 20*α*, 26-triol-3-O-*α*-L-rhamnopyranosyl-(1→2)-[*α*-L-rhamnopyranosyl-(1→4)]-*β*-D-glucopyranoside (Qin et al., 2012)
110	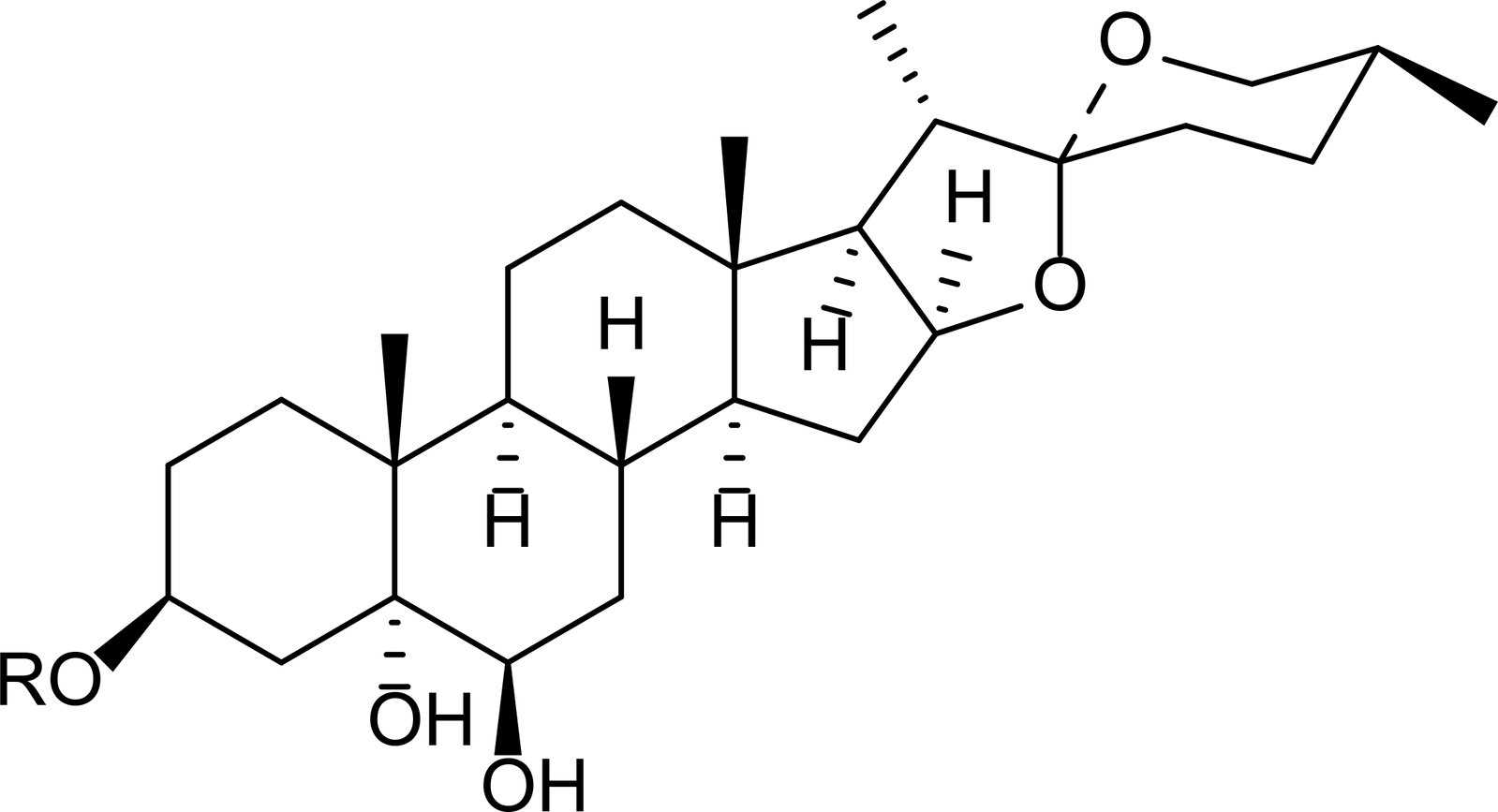	Api-(1→3)-[Rha-(1→2)]-Glc			(3*β*, 5*α*, 6*β*, 25R)-spirostane-3, 5, 6-triol-3-O-*β*-D-apiofuranosyl-(1→3)-[*α*-L-rhamnopyranosyl-(1→2)]-*β*-D-glucopy-ranosid (Wu et al., 2012a)
111		Rha-(1→2)-Glc			(3*β*, 5*α*, 6*β*, 25R)-spirostane-3, 5, 6-triol-3-O-*α*-L-rhamnopyranosyl-(1→2)-*β*-D-glucopyranosid (Wu et al., 2012a)
		R_1_	R_2_		
112	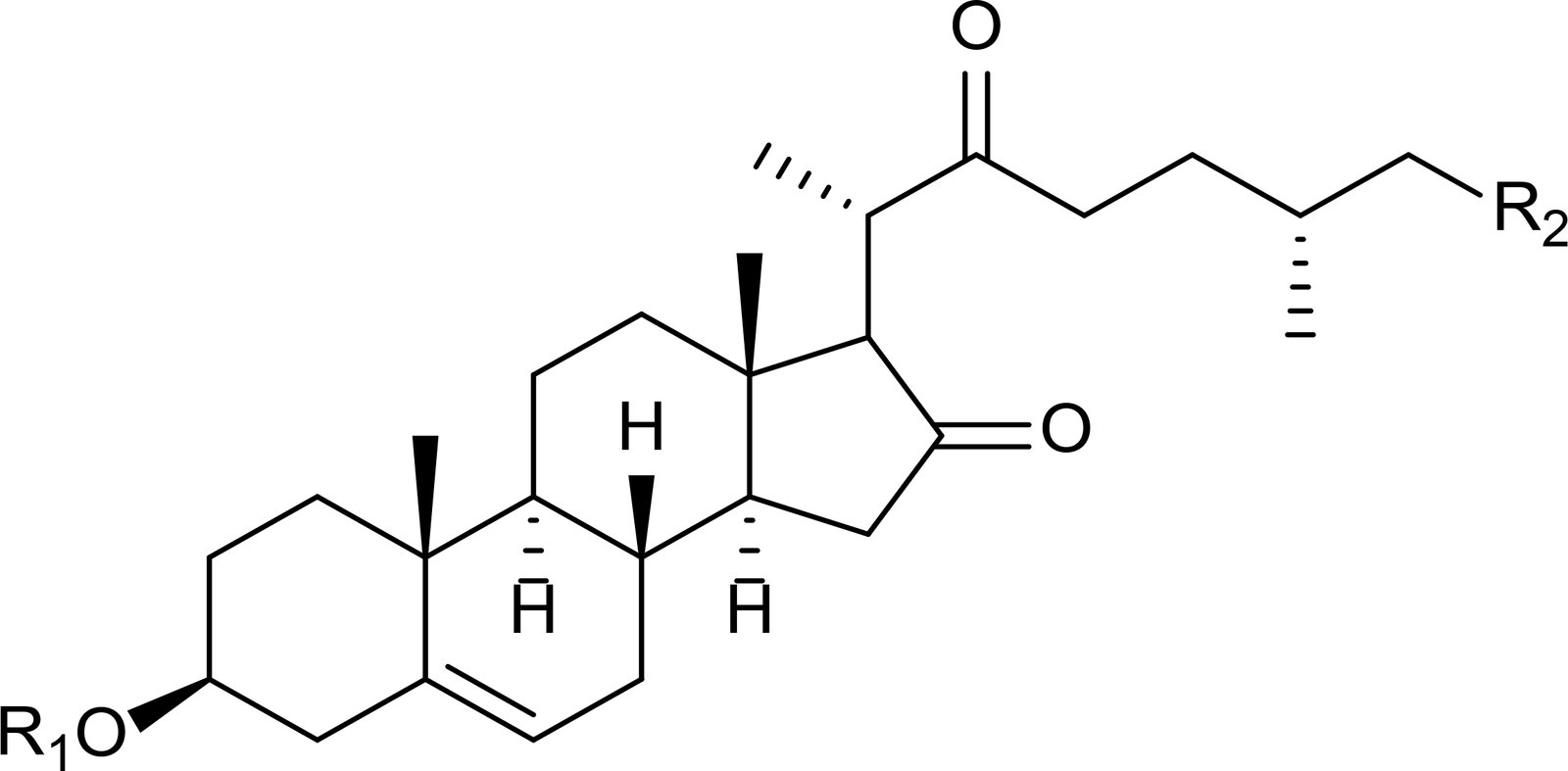	Rha-(1→4)-Rha-(1→4)-[Rha-(1→2)]-Glc	Glc		26-O-*β*-D-glucopyranosyl-kryptogenin-3-O-*α*-L-rhamnopyranosyl-(1→4)-*α*-L- rhamnopyranosyl-(1→4)-[*α*-L-rhamnopy-ranosyl-(1→2)]-*β*-D-glucopyranoside (Tao et al., 2020)
113	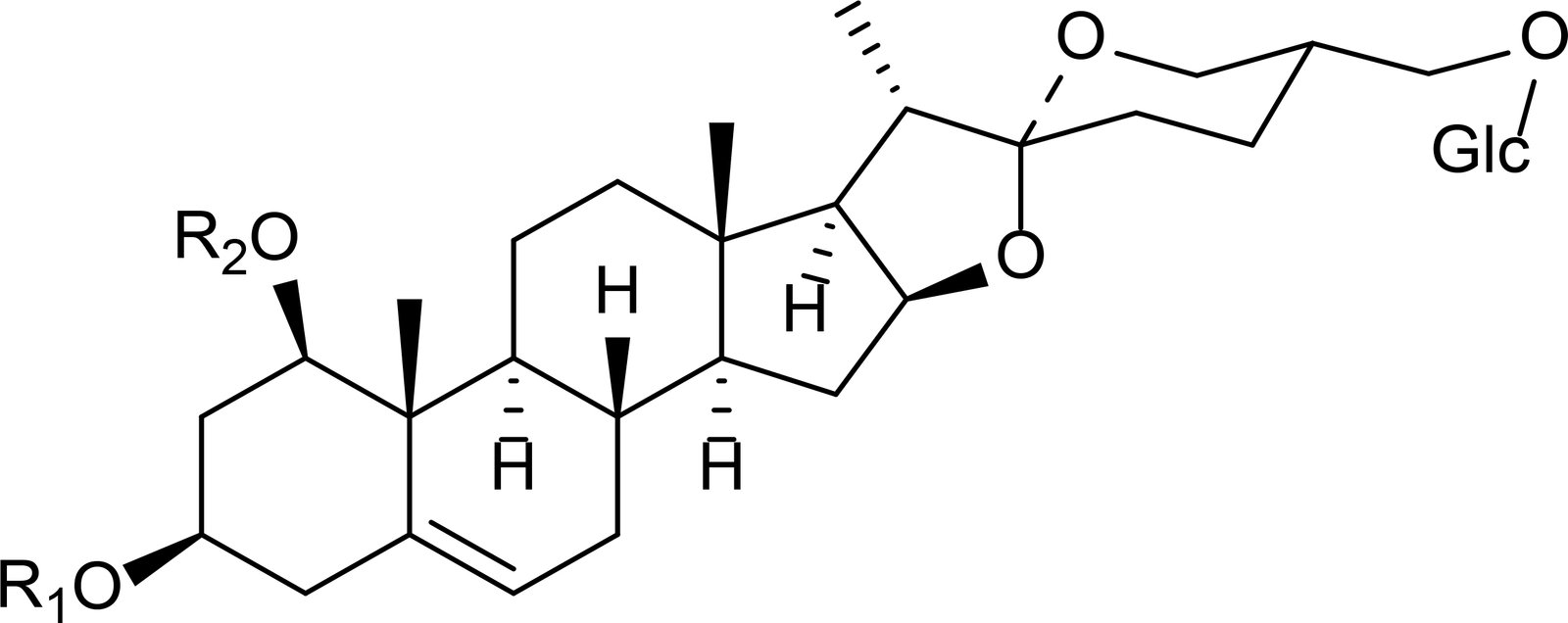	Rha-(1→2)-[Xyl-(1→4)]-Glc	H		chonglouoside SL-6 (Qin et al., 2012)
114	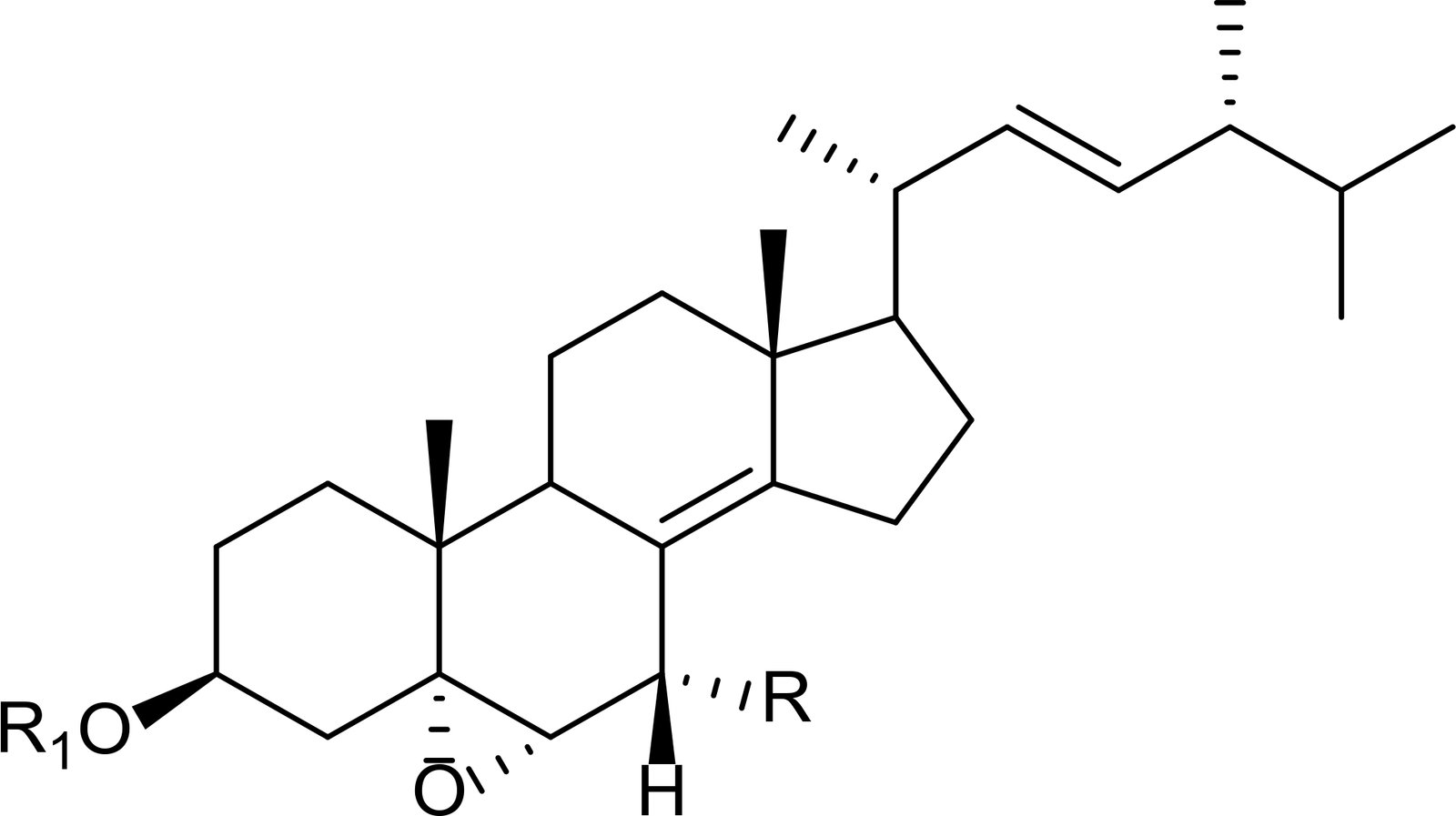	H	OH		5*α*, 6*α*-epoxy-(22E, 24R)-ergosta-8(14), 22-diene-3*β*, 7*α*-diol (Jiang et al., 2021)
115	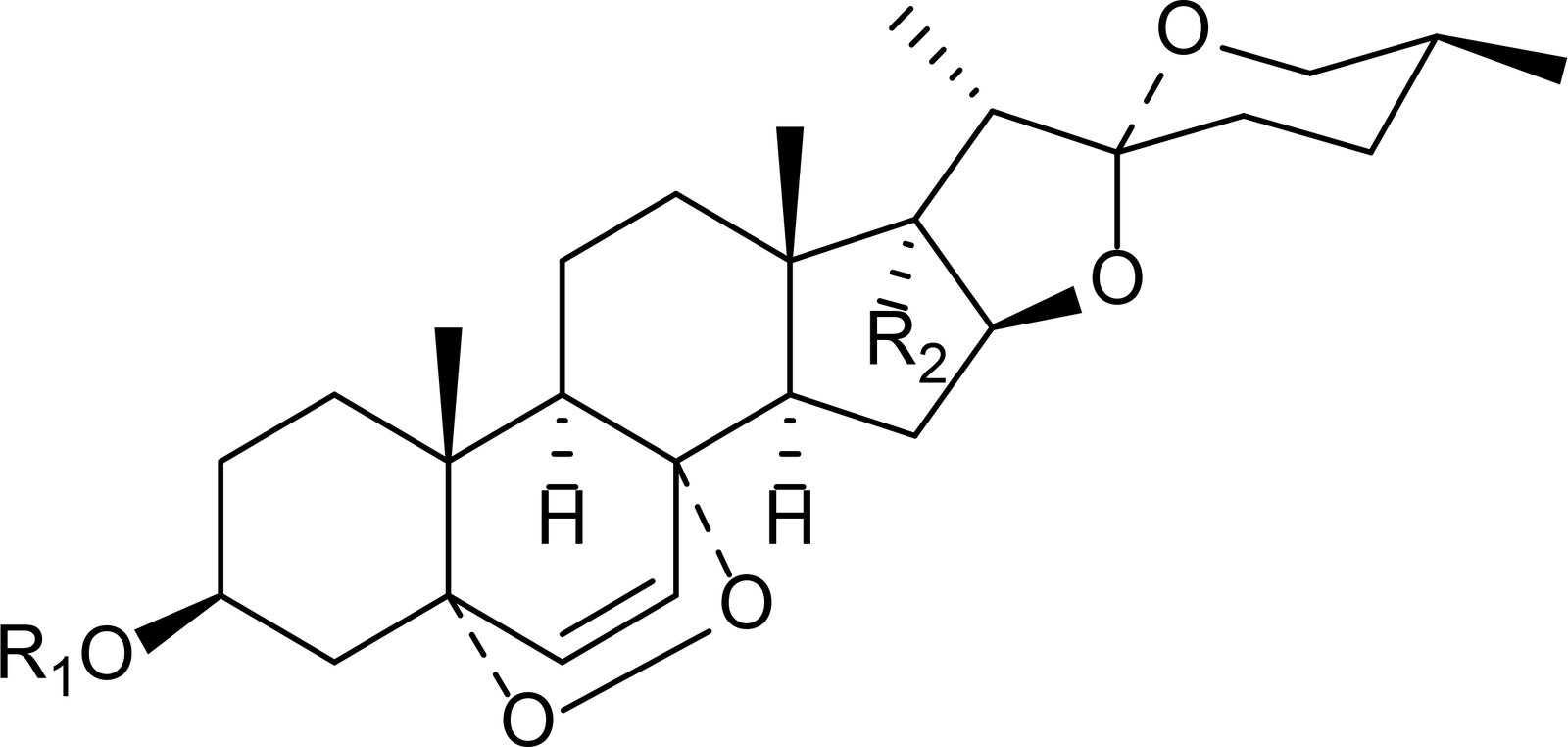	Rha-(1→2)-Glc	H		pariposide A (Wu et al., 2012b)
116		Api-(1→3)-[Rha-(1→2)]-Glc	H		pariposide B (Wu et al., 2012b)
117		Ara-(1→4)-[Rha-(1→2)]-Glc	H		pariposide C (Wu et al., 2012b)
118		Rha-(1→2)-Glc	OH		pariposide D (Wu et al., 2012b)
		R_1_	R_2_	R_2_	
119	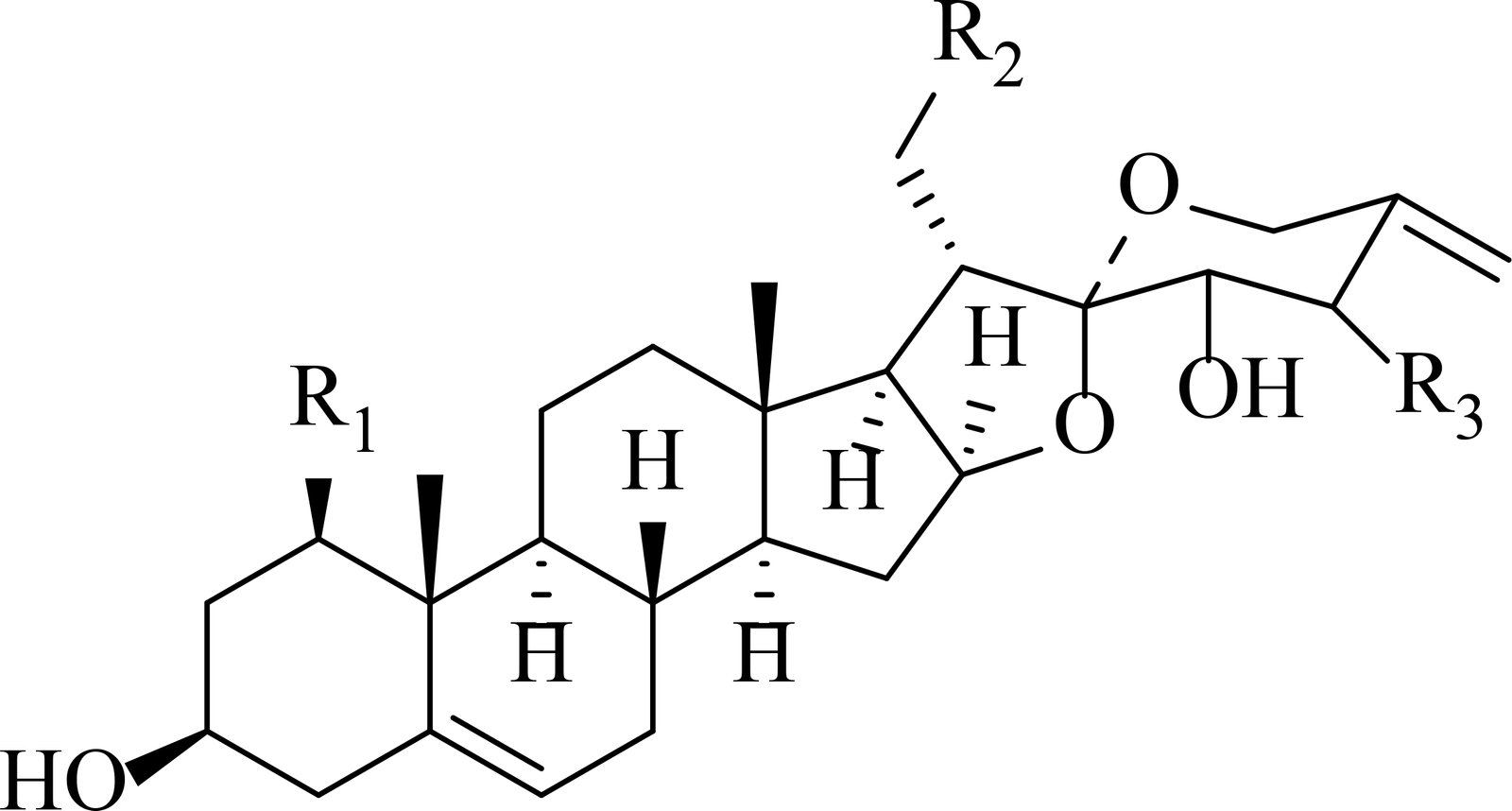	Xyl-(1→6)-Glc-(1→3)[Rha-(1→2)]-Glc	OH	O-Gal	24-O-*β*-D-galactopyranosyl(23S, 24S)-spirosta-5, 25(27)-diene-1*β*, 3*β*, 23, 24-tetrol-1--O-*β*-D-xylopyranosyl-(1→6)-*β*-D-glucopyranosyl-(1→3)-]*α*-L-rhamnopyranosyl-(1→2)]-*β*-D-glucopyranoside (Xu et al., 2007)
120		Rha-(1→2)-[Xyl-(1→3)]-Glc	O-Gal	Fuc	(23S, 24S)-24-O-*β*-D-fucopyranosyl-21-O-*β*-D-galactopyranosyl-1*β*, 3*β*, 21, 23, 24-pentahydroxyspirosta-5, 25(27)-diene-1-O-*α*-L-rhamnopyranosyl-(1→2)-]*β*-D-xylopyranosyl-(1→3)]-*β*-D-glucopyranoside(parisyunnanoside (Kang et al., 2012)
121	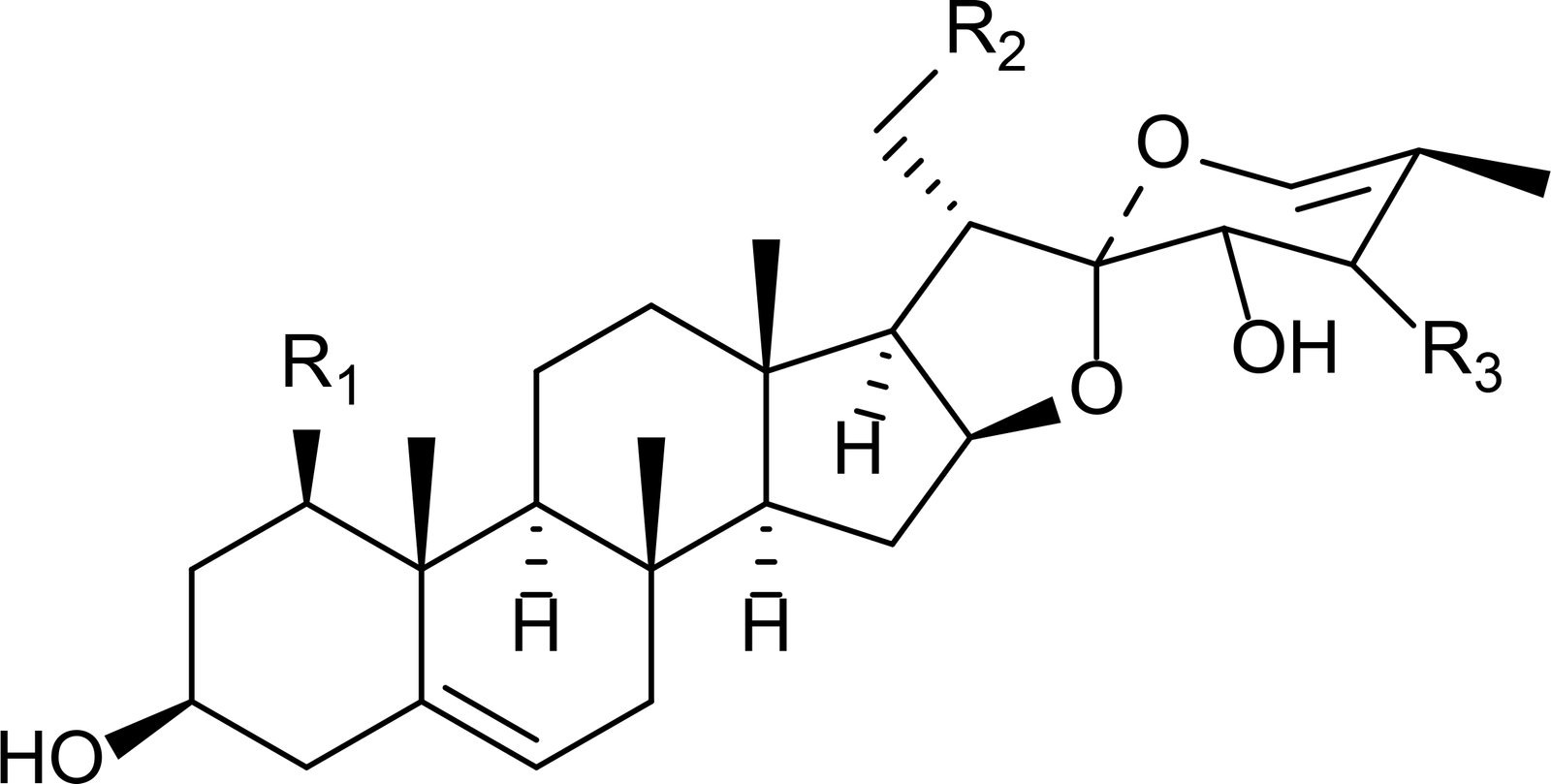	Rha-(1→2)-[Xyl-(1→3)]-Glc	Gal	Fuc	parisyunnanoside G e (Kang et al., 2012)
122		Rha-(1→2)-[Xyl-(1→3)]-Glc	OH	Fuc	parisyunnanoside H e (Kang et al., 2012)

### 4.3 Diversity of glycosyl moieties in steroidal saponins of *P*. *polyphylla*

Glycosylation is a key modification process in the biosynthesis of steroidal saponins, in which activated sugar donors are transferred to specific hydroxyl groups of steroidal aglycones under the catalysis of UDP-dependent glycosyltransferases (UGTs). This modification not only generates the remarkable structural diversity of steroidal saponins but also markedly influences their physicochemical properties, biological activities, stability, and water solubility (Shin and Oh, 2023; Zhang et al., 2022). As glycosylation is a major determinant of the structural diversity of steroidal saponins, systematically characterizing the composition and linkage patterns of glycosidic chains is essential for understanding their structural diversity and provides an important structural basis for elucidating the mechanisms underlying their biosynthetic diversification.

The glycosylation patterns of steroidal saponins in *P*. *polyphylla* exhibit remarkable structural diversity. Variations in the types, numbers, and linkage modes of glycosyl moieties collectively define the distinctive structural features of these compounds and confer diverse biological activities. From the perspective of glycosyl number, steroidal saponins in *P*. *polyphylla* can be categorized into aglycones without sugar moieties, as well as monosaccharide chains, disaccharide chains, trisaccharide chains, and tetra-saccharide chains, among which disaccharide, trisaccharide, and tetrasaccharide chains represent the predominant forms. Each category is associated with representative compounds; for example, polyphyllin V and polyphyllin VI possess disaccharide chains, polyphyllin I and polyphyllin III contain trisaccharide chains, whereas polyphyllin II and polyphyllin VII are characterized by tetrasaccharide chains. Regarding the types of sugar residues, the glycosyl moieties of steroidal saponins in *P*. *polyphylla* are mainly composed of Glc, Rha, and Ara, and may also include apiose (Api) and xylose (Xyl). The diverse combinations of these sugar units provide the structural basis for the extensive diversity of glycosidic chains.

**Figure 10:**
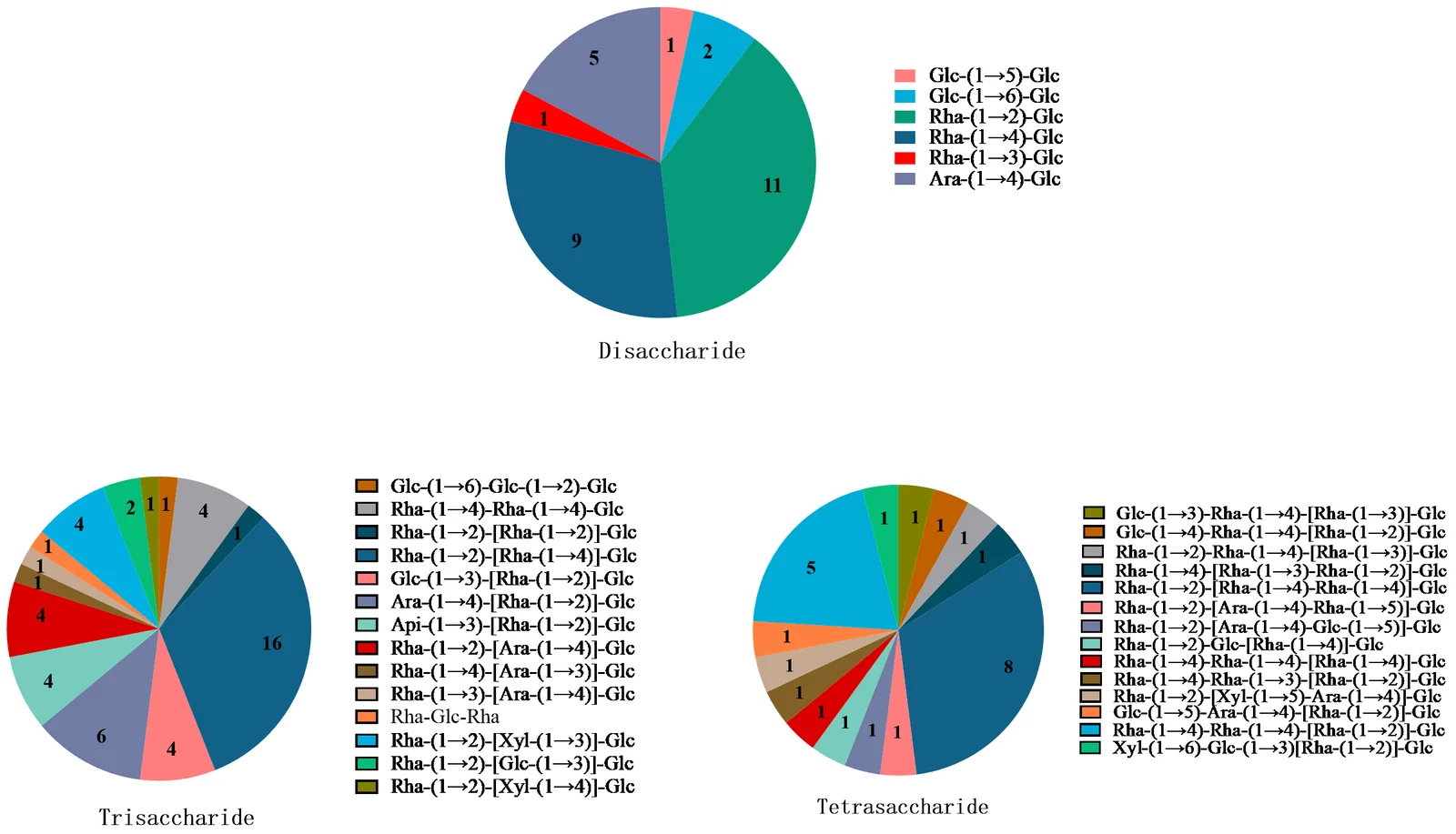
Summary of glycosidic linkage patterns of disaccharide, trisaccharide, and tetra-saccharide.

As shown in **Tables 1**–**7**, a statistical analysis of 122 steroidal saponins from *P*. *polyphylla* was conducted based on the linkage patterns of their glycosidic chains (**Table 8**; **Figure 10**). Monosaccharide chains are primarily composed of a single Glc unit, representing the simplest form of glycosides. Disaccharide chains mainly include linkage patterns such as Rha-(1→2)-Glc, Rha-(1→4)-Glc, and Ara-(1→4)-Glc, among which Rha-(1→2)-Glc is the most commonly observed structure in disaccharide-type steroidal saponins. The linkage patterns of trisaccharide chains are more complex and are predominantly characterized by branched structures in which a disaccharide side chain is attached to a monosaccharide backbone (Glc); for example, Rha-(1→2)-[Rha-(1→4)]-Glc represents the most common configuration. Tetrasaccharide chains exhibit the highest level of structural complexity, typically featuring Rha-centered composite structures that further connect with monosaccharides such as Glc, Ara, and Xyl to form diverse linkage patterns. Representative examples include Rha-(1→2)-[Rha-(1→4)-Rha-(1→4)]-Glc and Rha-(1→4)-Rha-(1→4)-[Rha-(1→2)]-Glc. These diverse linkage modes further enrich the structural diversity of glycosidic chains.

**Table 8:** Summary of glycosylation patterns of steroidal saponins in *P*. *polyphylla.*.

Number of glycosyl moieties	Glycosidic linkage sequence	Corresponding compound numbers		Total
–	–	1	diosgenin	12
		24	(25R)-spirost-5-en-3*β*, 7*β*-diol	
		25	(25R)-spirost-5-en-3*β*, 7*α*-diol	
		61	pennogenin	
		62	27-ol-pennogenin	
		63	27, 23*β*-diol-pennogenin	
		85	Paripolin H	
		86	chonglouoside SL-9	
		87	chonglouoside SL-10	
		106	22E-7*α*-methoxy-5*α*, 6*α*-epoxyergosta-8(14), 22-dien-3*β*-ol	
		107	20*β*-hydroxyecdysone	
		114	5*α*, 6*α*-epoxy-(22E, 24R)-ergosta-8(14), 22-diene-3*β*, 7*α*-diol	
Monosaccharide Chain	Glc	2	trillin	6
		26	chonglouoside SL-1	
		36	chonglouoside SL-17	
		81	Paripolin G	
		88	chonglouoside SL-11	
		89	chonglouoside SL-13	
Disaccharide Chain	Glc-(1→5)-Glc	47	paris saponin XI	1
	Glc-(1→6)-Glc	3	diosgenin-3-O-*β*-D-glucopyranosyl-(1→6)-*β*-D-glucopyranoside	2
		38	chonglouoside SL-18	
	Rha-(1→2)-Glc	4	polyphyllin V	11
		27	sansevierin A	
		49	trigofoenoside A	
		64	polyphyllin IV	
		91	26-O-*β*-D-glucopyranosyl-nuatigenin-3-O-*α*-L-rhamnopyranosyl-(1→2)-*β*-D-glucopyranoside	
		92	nuatigenin-3-O-*α*-L-rhamnopyranosyl-(1→2)-*β*-D-glucopyranoside	
		99	(25S)-spirost-5-en-3*β*, 25-diol-3-O-*α*-L-rhamnopyranosyl-(1→2)-*β*-D-glucopyranoside	
		103	(3*β*, 25R) -3-ol-spirost-5-en-7-one-3-O-*α*-L-rhamnopyranosyl-( 1→2) -*β*-D-glucopyranoside	
		111	(3*β*, 5*α*, 6*β*, 25R)-spirostane-3, 5, 6-triol-3-O-*α*-L-rhamnopyranosyl-(1→2)-*β*-D-glucopyranoside	
		115	pariposide A	
		118	pariposide D	
	Rha-(1→4)-Glc	5	diosgenin-3-O-*α*-L-rhamnopyranosyl-(1→4)-*β*-D-glucopyranoside	9
		28	disoseptemloside D	
		37	chonglouoside SL-2	
		44	Paripolin F	
		65	pennogenin-3-O-*α*-L-rhamnopyranosyl-(1→4)-*β*-D-glucopyranoside	
		82	(23S, 25S)-17*α*, 23, 27-trihydroxyspirost-5-en-3*β*-yl-O-*α*-L-rhamnopyranosyl-(1–4)-*β*-D-glucopyranoside	
		90	26-O-*β*-D-glucopyranosyl-nuatigenin-3-O-*α*-L-rhamnopyranosyl-(1→4)-*β*-D-glucopyranoside	
		93	abutiloside L	
		100	trigofoenoside A	
	Rha-(1→3)-Glc	6	polyphyllin C	1
	Ara-(1→4)-Glc	7	diosgenin-3-O-*α*-L-arabinofuranosyl-(1→4)-*β*-D-glucopyranoside	5
		29	(25R)-spirost-5-en-3*β*, 7*β*-diol-3-O-*β*-D-arabinofuranosyl-(1→4)-*β*-D-glucopyranoside	
		66	pennogenin-3-O-*α*-L-arabinofuranosyl-(1→4)-*β*-D-glucopyranoside	
		67	27-hydroxypennogenin 3-O-*α*-L-arabinofuranosyl-(1→4)-*β*-D-glucopyranoside	
		79	(3*β*, 17*α*, 25S)-spirost-5-ene-3, 17, 27-triol-3-O-*β*-D-arabinofuranosyl-(1→4)-*β*-D-glucopyranoside	
Trisaccharide Chain	Glc-(1→6)-Glc-(1→2)-Glc	8	diosgenin-3-O-*β*-D-glucopyranosyl-(1→6)-*β*-D-glucopyranosyl-(1→2)-*β*-D-glucopyranoside	1
	Rha-(1→4)-Rha-(1→4)-Glc	9	diosgenin-3-O-*α*-L-rhamnopyranosyl-(1→4)-*α*-L-rhamnopyranosyl-(1→4)-*β*-D-glucopyranoside	4
		46	Paripolin E	
		71	pennogenin-3-O-*α*-L-rhamnopyranosyl-(1→4)-*α*-L-rhamnopyranosyl-(1→4)-*β*-D-glucopyranoside	
		84	(23S, 25S)-17*α*, 23, 27-trihydroxyspirost-5-en-3*β*-yl-O-*α*-L-rhamnopyranosyl-(1 → 4)-*β*-D-gluco-pyranoside	
	Rha-(1→2)-[Rha-(1→2)]-Glc	51	parisaponin I	1
	Rha-(1→2)-[Rha-(1→4)]-Glc	10	polyphyllin III	16
		35	chonglouoside SL-16	
		39	chonglouoside SL-3	
		40	chonglouoside SL-4	
		41	borassoside B	
		45	paripolin D	
		54	26-O-*β*-D-glucopyranosyl-(25R)-22-methoxy-furost-5-en-3*β*, 26-diol- 3-O-*α*-L-rhamnopyranosyl-(1→2)-[*α*-L-rhamnopy-ranosyl (1→4)]-*β*-D-glucopyranoside	
		58	chonglouoside SL-20	
		72	polyphyllin B	
		83	(23S, 25S)-5-en-spirost-3*β*, 17*α*, 23*α*, 27-tetraol 3-O-*α*-L-rhamnopyranosyl-(1 → 4)-*α*-L-rhamnopyranosy1-(1 → 2)-*β*-D-glucopyranoside	
		95	26-O-*β*-D-glucopyranosyl-nuatigenin-3-O-*α*-L-rhamnopyranosyl- (1→2)-[*α*-L-rhamnopyranosyl-(1→4)]-*β*-D-glucopy-ranoside	
		96	26-O-*β*-D-glucopyranosyl 12*α*-hydroxynuatigenin 3-O-*α*-L-rhamnopyranosyl-(1 → 2)-[*α*-L-rhamnopyranosyl-(1 → 4)]-*β*-D-glucopyronoside	
		97	chonglouoside SL-15	
		101	25S-isonuatigenin-3-O-*α*-L-rhamnopyranosyl-(1→2)-[*α*-L-rhamnopyranosyl-(1→4)]-*β*-D-glucopyranoside	
		105	3-O-*β*-chacotriosyl kryptogenin 26-O-*β*-D-glucopyranoside	
		109	26-O-*β*-D-glucopyranosyl-(25R)-5, 22-diene-furost-5-en-3*β*, 20*α*, 26-triol-3-O-*α*-L-rhamnopyranosyl-(1→2)-[*α*-L-rhamnopyranosyl-(1→4)]-*β*-D-glucopyranoside	
	Glc-(1→3)-[Rha-(1→2)]-Glc	11	gracillin	4
		32	(25R)-spirost-5-en-3*β*, 7*β*-diol-3-O-*β*-D-glucopyranosyl-(1→3)-[*α*-L- rhamnopyranosyl-(1→2)]-*β*-D-glucopyranoside	
		69	pennogenin-3-O-*α*-L-rhamnopyranosyl-(1→2)-[*β*-D-glucopyranosyl-(1→3)]-*β*-D-glucopyranoside	
		108	(25R)-spirost-7(8)-ene-3*β*, 6*β*-diol-3-O-*β*-D-glucopyranosyl-(1→3)-[*α*-L-rhamnopyranosyl-(1→2)]-*β*-D-glucopyranoside	
	Ara-(1→4)-[Rha-(1→2)]-Glc	30	parisyunnanoside E	6
		31	parisyunnanoside D	
		52	parisyunnanoside A	
		59	parisyunnanoside B	
		70	paris saponin H	
		117	pariposide C	
	Api-(1→3)-[Rha-(1→2)]-Glc	12	diosgenin-3-O-*α*-L-rhamnopyranosyl-(1→2)-[*α*-L-apiofuranosyl-(1→3)]-*β*-D-glucopyranoside	3
		68	pennogenin-3-O-*α*-L-rhamnopyranosyl-(1→2)-[*β*-D-apiofuranosyl-(1→3)]-*β*-D-glucopyranoside	
		116	pariposide B	
	Rha-(1→2)-[Ara-(1→4)]-Glc	13	polyphyllin I	4
		42	dianchonglouoside B	
		53	26-O-*β*-D-glucopyranosyl-(25R)-22-methoxy-furost-5-en-3*β*, 26-diol-3-O-*α*-L-rhamnopyranosyl-(1→2)-[*β*-D-arabinofuranosyl-(1→4)]-*β*-D-glucopyranoside	
		80	dianchonglouoside A	
	Rha-(1→4)-[Ara-(1→3)]-Glc	14	diosgenin-3-O-*α*-L-rhamnopyranosyl-(1→4)-[*α*-L-arabinofuranosyl-(1→3)]-*β*-D-glucopyranoside	1
	Rha-(1→3)-[Ara-(1→4)]-Glc	15	diosgenin-3-O-*α*-L-rhamnopyranosyl-(1→3)-[*α*-L-arabinofuranosyl-(1→4)]-*β*-D-glucopyranoside	1
	Rha-Glc-Rha	33	dioseptemloside G	1
	Rha-(1→2)-[Xyl-(1→3)]-Glc	48	padelaoside B	4
		120	O-*β*-D-xylopyranosyl-(1→6)-*β*-D-glucopyranosyl-(1→3)-[*α*-L-rhamnopyranosyl-(1→2)]-*β*-D-glucopyranoside	
		121	parisyunnanoside G	
		122	parisyunnanoside H	
	Rha-(1→2)-[Glc-(1→3)]-Glc	50	protogracillin	2
		94	chonglouoside SL-12	
	Rha-(1→2)-[Xyl-(1→4)]-Glc	113	chonglouoside SL-6	1
Tetrasaccharide Chain	Glc-(1→3)-Rha-(1→4)-[Rha-(1→3)]-Glc	16	pariphyllin A	1
	Glc-(1→4)-Rha-(1→4)-[Rha-(1→2)]-Glc	17	diosgein-3-O-*β*-D-glucopyranosyl-(1→4)-*α*-L-rhamnopyranosyl-(1→4)-[*α*-L-rhamnopyranosyl-(1→2)]-*β*-D-glucopy-ranoside	1
	Rha-(1→4)-[Rha-(1→3)-Rha-(1→2)]-Glc	19	polyphyllin F	1
	Rha-(1→2)-[Rha-(1→4)-Rha-(1→4)]-Glc	20	polyphyllin II	8
		21	parisyunnanoside C	
		55	dichotomin	
		56	saponin Th	
		57	26-O-*β*-D-glucopyranosyl-(25R)-22-methoxy-furost-5-en-3*β*, 26-diol- 3-O-*α*-L-rhamnopyranosyl-(1→2)-[*α*-L-rhamnopy-ranosyl-(1→4)-*α*-L- rhamnopyranosyl(1→4)]-*β*-D-glucopyranosid	
		60	pseudoproto-Pb	
		73	polyphyllin VI	
		102	chonglouoside SL-5	
	Rha-(1→2)-[Ara-(1→4)-Rha-(1→5)]-Glc	22	reclinatoside	1
	Rha-(1→2)-[Ara-(1→4)-Glc-(1→5)]-Glc	23	loureiroside	1
	Rha-(1→2)-Glc-[Rha-(1→4)]-Glc	34	disoseptemloside E	1
	Rha-(1→4)-Rha-(1→4)-[Rha-(1→4)]-Glc	43	(3*β*, 25S)-spirost-5-ene-3, 27-diol-3-O-*α*-L-rhamnopyranosyl-(1→4)-*α*- L-rhamnopyranosyl-(1→4)-[*α*-L-rhamnopyranosyl-(1→2)]-*β*-D-glucopyranoside	1
	Rha-(1→4)-Rha-(1→3)-[Rha-(1→2)]-Glc	74	pennogenin-3-O-*α*-L-rhamnopyranosyl-(1→4)-*α*-L-rhamnopyranosyl-(1→3)-[*α*-L-rhamnopyranosyl-(1→2)]-*β*-D-glucopyranoside	1
	Rha-(1→2)-[Xyl-(1→5)-Ara-(1→4)]-Glc	75	pennogenin-3-O-*β*-D-xylopyranosyl-(1→5)-*α*-L-arabinofuranosyl-(1→4)-[*α*-L-rhamnopyranosyl-(1→2)]-*β*-D-glucopy-ranoside	1
	Glc-(1→5)-Ara-(1→4)-[Rha-(1→2)]-Glc	76	pennogenin-3-O-*β*-D-glucopyranosyl-(1→5)-*α*-L-arabinofuranosyl-(1→4)-[rhamnopyranosyl-(1→2)]-*β*-D-glucopyranoside	1
	Rha-(1→4)-Rha-(1→4)-[Rha-(1→2)]-Glc	77	polyphylloside III	5
		78	polyphylloside IV	
		98	chonglouoside SL-14	
		104	chonglouoside SL-19	
		112	26-O-*β*-D-glucopyranosyl-kryptogenin-3-O-*α*-L-rhamnopyranosyl-(1→4)-*α*-L- rhamnopyranosyl-(1→4)-[*α*-L-rhamnopy-ranosyl-(1→2)]-*β*-D-glucopyranoside	
	Xyl-(1→6)-Glc-(1→3)[Rha-(1→2)]-Glc	119	24-O-*β*-D-galactopyranosyl(23S, 24S)-spirosta-5, 25(27)-diene-1*β*, 3*β*, 23, 24-tetrol-1--O-*β*-D-xylopyranosyl-(1→6)-*β*-D-glucopyranosyl-(1→3)-]*α*-L-rhamnopyranosyl-(1→2)]-*β*-D-glucopyranoside	1

## 5 Biosynthetic pathway of steroidal saponins in *P*. *polyphylla*

**Figure 11:**
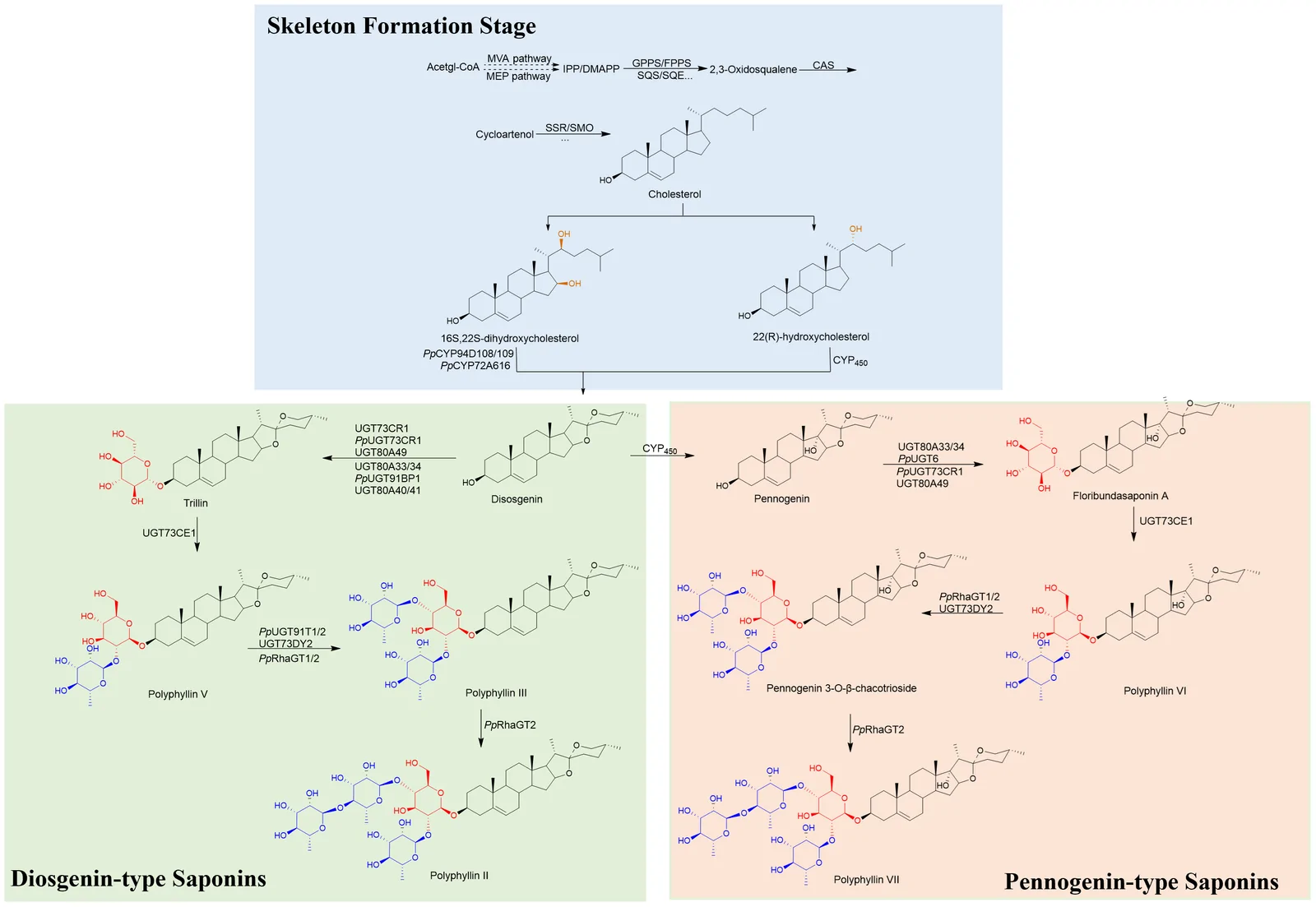
Predicted biosynthetic pathway of major steroidal saponins in *paris* species.

The biosynthesis of steroidal saponins in *P. polyphylla* is a complex process involving the coordinated action of multiple enzymes. It primarily encompasses the formation of upstream precursors and the construction of the core skeleton, as well as downstream modifications, including aglycone tailoring and glycosylation. To date, the core mechanisms underlying this biosynthetic process have been progressively elucidated through integrated multi-omics analyses, enzyme functional characterization, and heterologous reconstruction approaches (**Figure 11**).

### 5.1 Upstream biosynthetic pathway

Cholesterol, as a central precursor of steroidal saponins, is synthesized through a process that can be broadly divided into two stages. First, isopentenyl diphosphate (IPP) and its isomer dimethylallyl pyrophosphate (DMAPP) are generated via the mevalonate (MVA) pathway and the methylerythritol phosphate (MEP) pathway, respectively, and subsequently converted into 2, 3-oxidosqualene through a series of enzymatic reactions. In the second stage, 2, 3-oxidosqualene is further catalyzed by a series of enzymes to produce cholesterol/β-sitosterol (Li et al., 2023).

The MVA pathway occurs in the cytosol and mitochondria, using acetyl-CoA as the substrate. Through the sequential catalysis of enzymes such as acetyl-CoA acetyltransferase (ACAT), 3-hydroxy-3-methylglutaryl-CoA synthase (HMGS), and mevalonate kinase (MVK), it leads to the formation of IPP. In contrast, the MEP pathway takes place in plastids, where glyceraldehyde-3-phosphate and pyruvate serve as the initial substrates. This pathway involves key enzymes including 1-deoxy-D-xylulose-5-phosphate syn-thase (DXS), 1-deoxy-D-xylulose-5-phosphate reductoisomerase (DXR), 2-C-methyl-D-erythritol-2, 4-cyclodiphosphate synthase (MDS), and (E)-4-hydroxy-3-methylbut-2-enyl diphosphate reductase (HDR), ultimately producing IPP/DMAPP (Li et al., 2023; Yang et al., 2019). Subsequently, IPP and DMAPP are converted into farnesyl diphosphate (FPP) under the catalysis of geranyl diphosphate synthase (GPPS) and farnesyl diphosphate synthase (FPPS). FPP is then condensed by squalene synthase (SQS) to form squalene, which is further epoxidized by squalene epoxidase (SQE) to yield 2, 3-oxidosqualene. Finally, 2, 3-oxidosqualene is cyclized by cycloartenol synthase (CAS) to produce cycloartenol, a key precursor of plant sterols. Cycloartenol subsequently undergoes multiple modification steps mediated by enzymes such as sterol side chain reductase (SSR), C-4 sterol methyl oxidase (SMO), cyclopropyl sterol isomerase (CPI), and sterol C-14 demethylase (CYP51G). These reactions involve progressive demethylation and double-bond rearrangement, ultimately leading to the formation of cholesterol (Guo et al., 2021; Yin et al., 2023).

### 5.2 Downstream biosynthetic pathway

The downstream stage of steroidal saponin biosynthesis begins with cholesterol as the initial substrate, which undergoes aglycone modifications to form various steroidal sapogenins. Subsequently, these steroidal sapogenins are subjected to sequential and ordered glycosylation reactions catalyzed by UDP-glycosyltransferases, ultimately yielding steroidal saponins with diverse glycosidic moieties.

#### 5.2.1 Aglycone modification stage

Cholesterol is initially subjected to specific catalysis by key enzymes of the cytochrome P450 (CYP450) family, during which hydroxylation and cyclization reactions occur at critical side-chain positions such as C-16, C-22, and C-26, leading to the formation of core sapogenins including diosgenin and pennogenin (Werck-Reichhart et al., 2024). Studies have demonstrated that CYP450 monooxygenases play a pivotal role in this process. Among them, *Pp*CYP90B27 specifically catalyzes the hydroxylation at the C-22 position of cholesterol to produce 22(R)-hydroxycholesterol, representing the first hydroxylation step in the biosynthesis of steroidal sapogenin (Yin et al., 2018). Further investigations have revealed that the formation of diosgenin involves the coordinated action of two classes of CYP450 enzymes. Initially, *Pp*CYP90G4 catalyzes the hydroxylation of cholesterol at the C-16 and C-22 positions to generate the intermediate 16S, 22S-dihydroxycholesterol; this step serves as both the initiating reaction and the rate-limiting step in diosgenin biosynthesis. Subsequently, under the catalysis of *Pp*CYP94D108/109 and *Pp*CYP72A616, hydroxylation occurs at the C-27 position of the intermediate, which further triggers an intramolecular cyclization between the C-27 and C-22 hydroxyl groups to form a spiroketal structure, ultimately yielding diosgenin (Christ et al., 2019).

#### 5.2.2 Glycosylation modification stage

Following the formation of steroidal sapogenins, the glycosylation process is mediated by UGTs, which catalyze the attachment of diverse sugar moieties to specific positions of the sapogenin back-bone, ultimately generating a wide variety of structurally distinct steroidal saponins. During the initial glycosylation step, multiple functionally specialized UGTs have been identified in *Paris* species. Among them, UGT73CR1 and UGT80A33/34 exhibit distinct catalytic characteristics in terms of substrate specificity and substrate promiscuity, respectively; UGT73CR1 primarily catalyzes glucosylation at the C-3 position, whereas UGT80A33/34 can broadly recognize steroidal sapogenin scaffolds (Song et al., 2022). Further studies have revealed that members of the UGT73C subfamily (e.g., *Pp*UGT6) specifically catalyze the initial glycosylation of pennogenin (He et al., 2023b). Enzymes such as UGT91BP1 exhibit strict selectivity toward specific substrates and sugar donors (UDP-glucose), enabling the selective catalysis of the first glycosylation step of diosgenin (Li et al., 2025). In addition, certain UGTs (e.g., UGT73CR1 and UGT80A49) are capable of acting on both diosgenin and pennogenin, mediating C-3 glycosylation to produce trillin and floribundasaponin A (He et al., 2023a; Hua et al., 2022).

During glycan chain elongation, multiple UGTs function cooperatively to enable stepwise modifications. UGT80A40/41 initially catalyze glucosylation at the C-3 position of diosgenin and pennogenin, producing trillin and floribundasaponin A, respectively. Subsequently, UGT73CE1 mediates 2′-O-rhamnosylation to generate disaccharide chains, namely polyphyllin V and polyphyllin VI. Finally, UGT91AH1/2/3 catalyze 6′-O-glucosylation, resulting in the formation of trisaccharide structures (Chen et al., 2023). Mean-while, UGTs such as PpUGT91T1/2, UGT73DY2, PpUGT91AH7, and *Pp*RhaGT1/2 are also involved in glycosylation at different positions, further enriching the structural diversity of sugar chains in steroidal saponins (Xie et al., 2025; Xu, 2024; Zhao et al., 2025; Zhou et al., 2025).

## 6 Conclusions and perspectives

This review systematically summarizes the research progress on the glycosidic linkage patterns and biosynthetic pathways of steroidal saponins in *P*. *polyphylla*. A total of 122 steroidal saponins have been identified in this species. Their aglycone structural characteristics, chemical classifications, and glycosylation patterns were comprehensively analyzed. Based on differences in aglycone structures, these saponins can be categorized into diosgenin-type, protodiosgenin-type, pseudoprotodiosgenin-type, pennogenin-type, nuatigenin-type, isonuatigenin-type, and other minor types, among which certain structural relationships are evident. The sugar moieties constituting these steroidal saponins are predominantly Glc, Rha, and Ara. In multi-glycosidic saponins, diverse glycosidic linkage patterns can arise even with the same number of sugar units, while a single linkage pattern may correspond to multiple distinct sapogenin backbones. The combinatorial diversity between sugar moieties and aglycones, together with structural variations such as substitution and modification of sugar chains, collectively contributes to the remarkable structural diversity of steroidal saponins in *P*. *polyphylla*. This structural complexity underlies the wide range of biological activities exhibited by these compounds.

Furthermore, the systematic analysis of the biosynthetic pathway presented in this review provides a plausible biosynthetic explanation for the recent changes in the quality control markers of Paris medicinal materials. The Chinese Pharmacopoeia of 2020 edition (Commission C.P, 2020) and 2025 edition (Commission C.P, 2025) designates polyphyllin I, II, and VII as quality control markers, whereas polyphyllin VI, which was included in the 2015 edition (Commission C.P, 2015), has been removed. Related studies have shown that polyphyllin VI exhibits a low detection rate and low content in *P*. *polyphylla* across different harvest periods, geographical origins, and growth years, and in some cases is not detected at all (Huang et al., 2017; Zhao et al., 2023). In conjunction with the findings of this study, it can be inferred that the low abundance of polyphyllin VI is closely associated with its role as an intermediate in the biosynthetic pathway of steroidal saponins. Specifically, this com-pound is not only a product of pennogenin glycosylation but can also be further catalyzed by UGTs to form steroidal saponins with trisaccharide and tetrasaccharide chains. Consequently, its content remains relatively low across different tissues of *P*. *polyphylla*. Nevertheless, this biosynthetic interpretation remains hypothetical and requires further validation through isotope-labeling experiments, enzyme kinetic analyses, and quantitative metabolic flux studies.

Despite the diverse pharmacological activities exhibited by steroidal saponins from *P*. *polyphylla*, substantial challenges remain in translating these compounds into clinically applicable products. Most of the reported antitumor, anti-inflammatory, antimicrobial, and hemostatic activities are currently supported by biochemical assays, cell-based studies, and animal experiments, whereas high-quality controlled clinical evidence remains limited. Furthermore, considerable variations in compound purity, experimental models, routes of administration, and dosing regimens among different studies hinder direct comparison of the reported findings and impede the establishment of reliable structure-activity relationships. Pharmacokinetic properties also represent a major barrier to their clinical translation (Lu et al., 2020; Wang et al., 2019). Future translational research should therefore focus on systematically elucidating the dose-exposure-response relationships, *in vivo* metabolic fate, tissue distribution, therapeutic window, chronic and reproductive toxicity, potential drug–drug interactions, and the influence of the gut microbiota on the pharmacological effects and biotransformation of steroidal saponins.

Meanwhile, the sustainable utilization of *P*. *polyphylla* resources continues to face significant challenges. Owing to its long growth cycle, increasing market demand, and prolonged overexploitation of wild populations, natural resources have undergone continuous depletion. Although artificial cultivation can partially alleviate the pressure on wild resources, substantial variations in steroidal saponin accumulation resulting from differences in genetic background, environmental conditions, and cultivation practices remain major obstacles to ensuring the consistent quality of medicinal materials. Therefore, the sustainable utilization of *P*. *polyphylla* requires an integrated strategy encompassing both *in situ* and *ex situ* conservation of wild germplasm resources, the identification and breeding of elite germplasm, as well as the establishment of standardized propagation, cultivation, and harvesting systems.

With respect to biosynthetic studies, substantial progress has been made in elucidating the chemical constituents and biosynthetic pathways of steroidal saponins in *P*. *polyphylla*. Nevertheless, several important challenges remain. Key enzymes involved in certain sapogenin transformation steps have yet to be fully characterized, including the C17α-hydroxylase responsible for the conversion of diosgenin to pennogenin. In addition, the enzymatic mechanisms governing the elongation of complex glycosidic chains remain to be clarified. Although recent advances in the identification and engineering of glycosyltransferases have enabled the heterologous reconstruction of several steroidal trisaccharides, the production yields achieved to date remain far below the levels required for economically viable industrial applications (Zhao et al., 2025).

Future research should focus on further elucidating the biosynthetic mechanisms of steroidal saponins, with particular emphasis on the functional characterization of key enzymes involved in sapogenin modification and glycosylation, especially cytochrome P450 enzymes and UDP-dependent glycosyltransferases (UGTs). The development of efficient heterologous expression systems for the *de novo* biosynthesis of steroidal saponins from *P*. *polyphylla* represents a promising strategy for sustainable production. This approach could not only alleviate pressure on wild resources but also enable the rational design of glyco-syltransferases with tailored substrate specificities, through the identification of key biosynthetic genes, to generate novel steroidal saponin derivatives with enhanced pharmacological activities. Furthermore, the deep integration of metabolic engineering and fermentation engineering to establish scalable production platforms-based on microbial cell factories or plant suspension cell cultures-will provide new strategies for the sustainable utilization of *Paris* germplasm resources and for quality regulation of its medicinal products (Rawat et al., 2023a).
